# Brain-to-brain entrainment: EEG interbrain synchronization while speaking and listening

**DOI:** 10.1038/s41598-017-04464-4

**Published:** 2017-06-23

**Authors:** Alejandro Pérez, Manuel Carreiras, Jon Andoni Duñabeitia

**Affiliations:** 1 BCBL – Basque Center on Cognition Brain and Language, 20009 Donostia, Spain; 2 Ikerbasque, Basque Foundation for Science, 48011 Bilbao, Spain; 3 Departamento de Filología Vasca, EHU/UPV, 48015 Bilbao, Spain

## Abstract

Electroencephalographic hyperscanning was used to investigate interbrain synchronization patterns in dyads of participants interacting through speech. Results show that brain oscillations are synchronized between listener and speaker during oral narratives. This interpersonal synchronization is mediated in part by a lower-level sensory mechanism of speech-to-brain synchronization, but also by the interactive process that takes place in the situation per se. These results demonstrate the existence of brain-to-brain entrainment which is not merely an epiphenomenon of auditory processing, during listening to one speaker. The study highlights the validity of the two-person neuroscience framework for understanding induced brain activity, and suggests that verbal information exchange cannot be fully understood by examining the listener’s or speaker’s brain activity in isolation.

## Introduction

Human verbal communication involves an exchange of information between two or more people in order to convey and receive intended meanings through speech, with speech production and speech perception the two constitutive parts of the process. To be effective, communication requires the ability to deliver a verbal message and the capacity to comprehend the message. Speech perception is achieved by coupling between the ongoing rhythmic neural activity of the listener and the quasi-rhythms of the speech signal^1–3^. This phenomenon is called brain entrainment to speech, and supports native speech intelligibility^4–6^. Interestingly, entrainment also seems to take place during speech production, as the neural activity of a speaker correlates with the amplitude envelope of the sound of their own utterances^7^. Thus, there is a tight relationship between the neural oscillatory activity of the listener and the physical properties of the perceived message, but also between the neural activity of the speaker and the verbal output being produced. In the current study, the question under investigation is whether these effects are independent of each other, or whether the oscillatory patterns of speaker and listener reflect a form of mutual dependency.

Evidence from prior research indicates that understanding the verbal production of others involves some form of neural coupling not only with the input signal, but also with the speaker’s neural system^8, 9^, and that electroencephalographic (EEG) inter-brain synchronizations are tightly linked to speech synchronizations between interlocutors^10^. Studies have also shown increased EEG interbrain synchronization associated with effective social coordination^11, 12^, which is highly relevant because verbal communication is a cooperative process that requires individuals to actively coordinate their thoughts in a dynamic and adaptive way. Thus, not only does brain oscillatory activity entrain to speech, but it probably becomes synchronized between the listener and the speaker due to (*i*) the shared sensory stimulus (speech signal), and (*ii*) the verbal communication itself (social interaction). We hypothesize the existence of brain-to-brain entrainment during a communicative scenario based on verbal interaction. This brain-to-brain entrainment would imply that the neural oscillators from two autonomous human brains interact (expressed via synchronization), while individuals engage in a verbal exchange.

This hypothesis was tested in the current study by investigating whether brain-to-brain synchronization appears when two persons exchange verbal narratives (oral narrative, hereafter). We used an experimental set-up which is in line with the *two-person neuroscience* (2PN) methodological and conceptual framework^13^. EEG activity was recorded simultaneously from a pair of subjects as they engaged in interchanging verbal information (15 dyads in total). The simultaneous measurement of neural activity from an interacting dyad is a technique known as hyperscanning^14^. Specifically, hyperscanning was performed while one component of the dyad listened to the other’s oral narrative (i.e. listener and speaker roles, respectively) without interpersonal visual contact. Participants alternated between the roles of speaker and listener. This setting can be considered a close-to-natural scenario since it resembles a telephone conversation in which pairs of persons hear but do not see each other. Enhancement in the interbrain synchronization patterns was evaluated by comparing the oral narrative recordings against a surrogate dataset obtained from pairing speaker and listener of the same dyad in non-time-corresponding moments of the ‘conversation’. Also, by using multiple linear regression modelling we assessed for the brain-to-brain entrainment not mediated by the speech-to-brain entrainment. Our results shed light on inter-related brain functions and the neural bases of language as social action, as well as highlighting the importance of the interactive nature of communication between individual brains.

## Results

### Behavior

The listeners’ attention was evaluated at the end of the interchange of oral narratives by asking each participant to complete a questionnaire containing questions on each of the topics on which the dyads constructed the discourse. Participants accurately responded on average to 90.5% of the 30 questions (6 questions on each of the 5 topics), demonstrating that the listeners were paying careful attention to the speakers’ narratives during the experiment (mean: 27, SD: 2.2).

### Brain-to-brain synchronization

**Figure 1:**
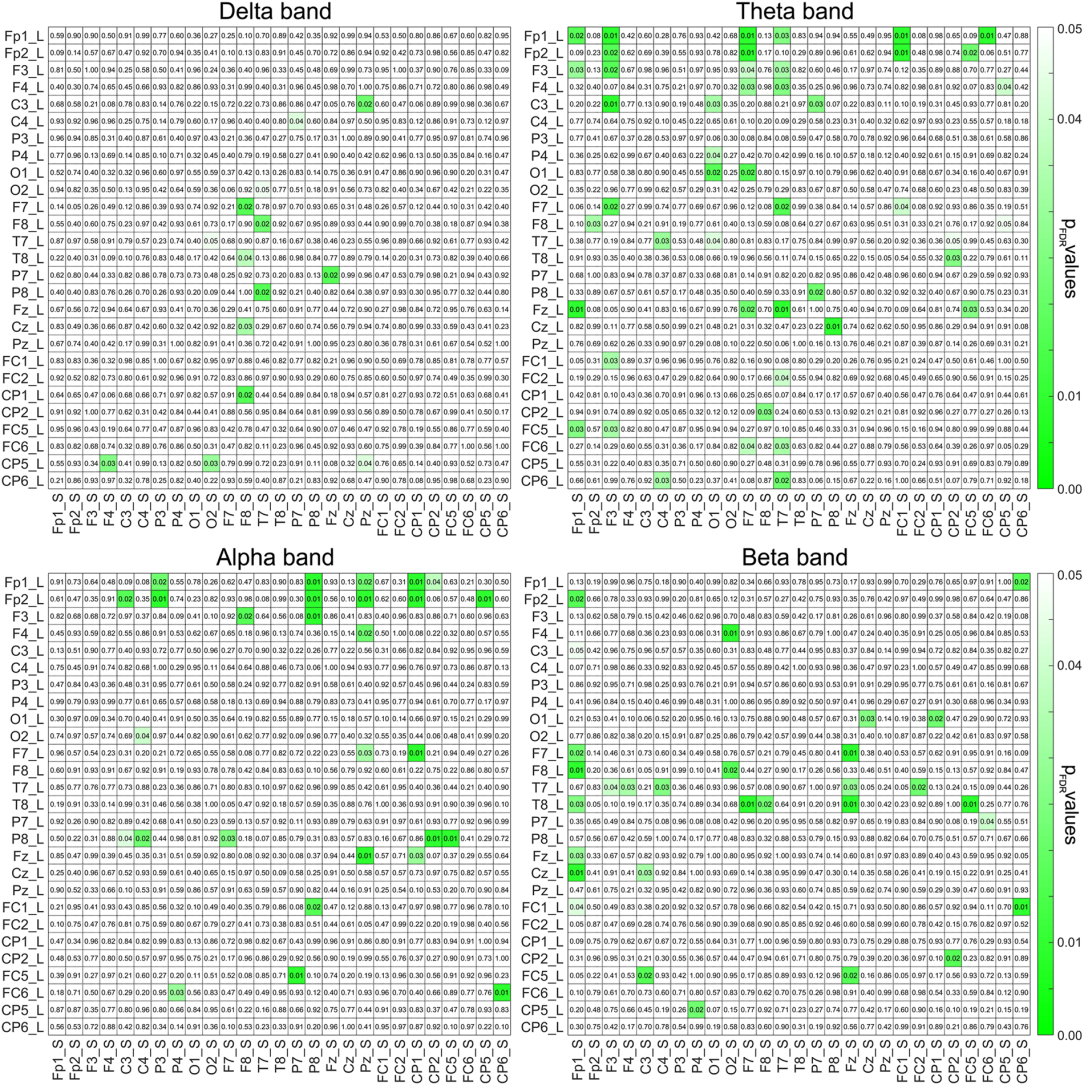
Group-level statistical results from the brain-to-brain synchronization analysis at the different frequency bands. The matrices contain the FDR-corrected p values from nonparametric permutation tests (rounded) between each channel of the Listener and each channel of the Speaker. Statistically significant coupling (PLV) between listeners and speakers during oral narratives is indicated by coloring in green the corresponding cells with p_FDR_ < 0.05. Rows represent the electrodes of the Listener and columns the electrodes of the Speaker. Significant interbrain synchronization, as compared to the surrogate dataset, is evident with different topographical patterns for delta, theta, alpha and beta frequency bands.

To compute brain-to-brain synchrony between Speaker and Listener we employed a phase synchrony measure known as phase locking value (PLV), which was calculated for all Listener/Speaker pair combinations of EEG signals (i.e. 27 × 27 electrodes) and then compared to a surrogate dataset to assess for the statistical significance of the degree of coupling or co-variations in their neural activity (see Statistical Analysis section). Statistically significant increases in PLV between the Listener and the Speaker appeared at the four frequency bands analyzed (delta, theta, alpha and beta), when compared to the surrogate data. After correcting for multiple comparisons across bands (4) and electrode pairs combinations (729) using a false discovery rate (FDR) of q < 0.05, the interbrain synchronization was enhanced at 123 electrode pairs: 14 in delta band, 49 in theta, 28 in alpha and 32 pairs in beta. Figure 1 show these patterns of interbrain synchronization in a matrix indicating the exact electrode pairs (Listener/Speaker) at which the enhancement occurred. Figure 1 also provides the (rounded) p values (FDR corrected, p_FDR_) for each electrode pair. The corresponding statistical values are in Supplementary Figure 1 (all df = 29). There is a differential topographical distribution of the effect for each frequency band, and only 3 electrode pairs showed effects across two or more bands. This indicates that the interbrain synchronization is not topographically constrained across bands or driven by the same electrodes.

These results demonstrate the existence of a brain-to-brain entrainment during oral narrative that could be due to the shared speech signal and/or the verbal communication itself. The following analyses are intended to determine whether the brain-to-brain entrainment is mediated (at least in part) by a lower-level sensory mechanism of speech-to-brain synchronization.

### Brain-audio envelope synchronization

**Figure 2:**
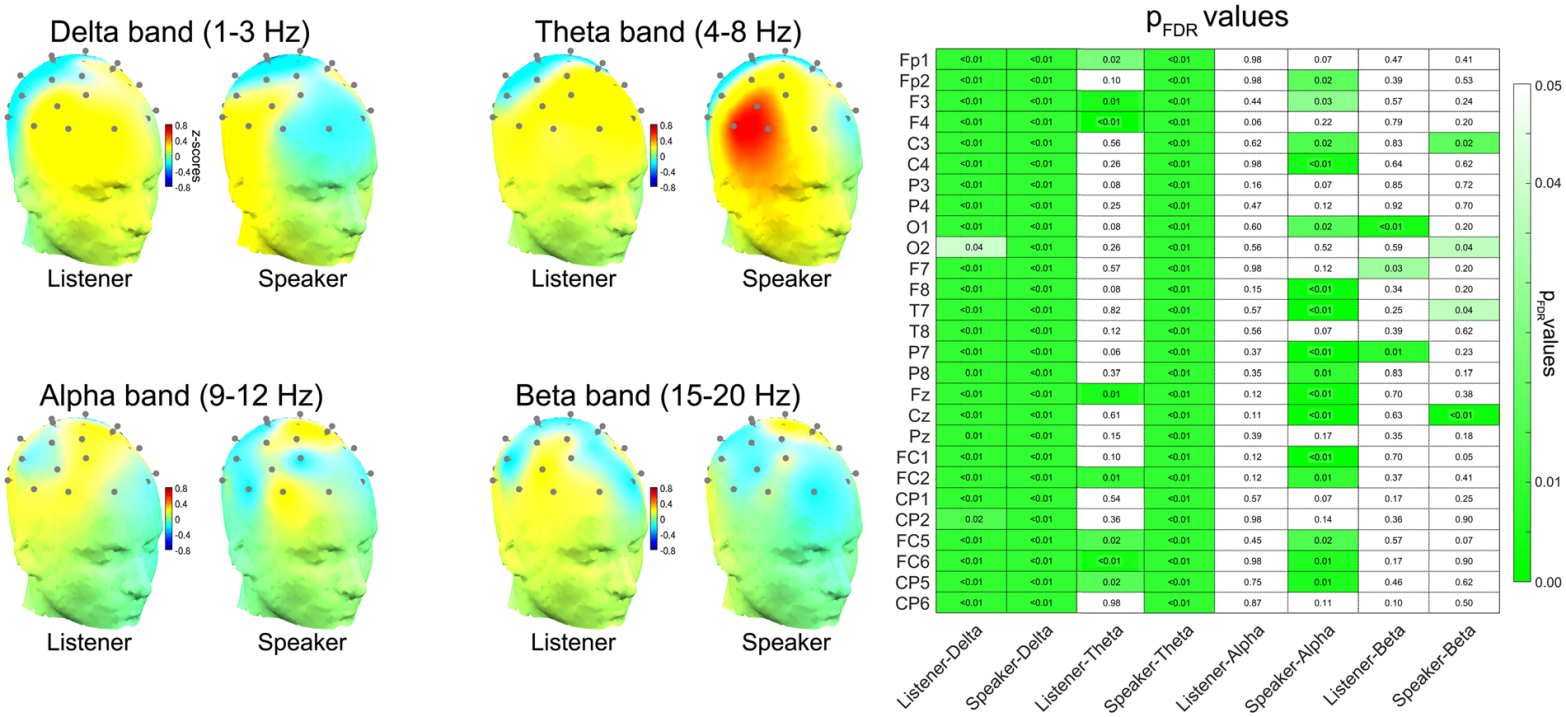
Brain entrainment to the narratives for listeners and speakers. The topological plots show the scalp distribution of the mean differences across participants in synchronization (PLV’s z-scores) between the real and the surrogate data at each frequency band. The different colors represent the scale from −0.8 to 0.8. The gray dots on the heads represent the location of the electrodes. The statistical results from the brain-audio envelope synchronization analysis at the different frequency bands are presented on the right. The matrix contains the FDR-corrected p values from nonparametric permutation tests (rounded) between each channel of the Listener/Speaker and the speech signal. Statistically significant brain-to-speech coupling (PLV) while listening or producing speech is indicated by coloring in green the corresponding cells where p_FDR_ < 0.05. Rows represent the electrodes and columns represent the role-frequency band. Significant brain-to-speech synchronization, as compared to the surrogate dataset, is more evident in the slow frequencies of delta and theta and more for the Speaker than for the Listener.

In order to determine the brain-to-brain entrainment mediated by speech-to-brain entrainment we first investigated the precise bands and sites showing statistically significant increases in phase synchronization (PLV) between the EEG signals and the amplitude envelopes of speech signals, again as compared to surrogate data. Increases in synchronization (i.e. brain-audio envelope synchronizations) were examined separately for the Listener and the Speaker. The heads in the left panel of Fig. 2 show the topographical distribution of the mean difference between the synchronization in the real and the surrogate data. This qualitative assessment highlights the different topographical patterns for Listeners and Speakers in the theta band, with the Speakers showing larger differences between real and surrogate data at right temporal sites. Figure 2 also shows the p_FDR_ values of the comparison for each electrode for both roles and frequency bands. A table with the statistics of these comparisons is included in Supplementary Figure 2 (all df = 29). After FDR correction (q < 0.05) across role-frequency bands (4) and electrodes (27), in the case of the delta band, neural entrainment to the amplitude envelope was found for both the Listener and the Speaker at all electrodes. In the case of theta, the effect was generalized across all electrodes for the Speakers while it was distributed fronto-centrally across 8 electrodes for the Listeners. In the case of alpha, neural entrainment to speech was evident only for the Speaker, who showed an effect across 16 electrodes, while no effect was observed for the Listener. In the case of beta band, the effects are circumscribed to a few electrodes for both the Listener and the Speaker (3 and 4, respectively).

These results demonstrate brain entrainment to speech while perceiving and producing oral narratives. This entrainment is particularly stronger at the low frequencies and it is increased and more generalized in the case of the Speaker as compared to the Listener. The following analysis was carried out to determine whether the brain-to-brain entrainment pattern could have been mediated or boosted by the Listener/Speaker’s brain entrainment to speech.

### Multiple linear regression modelling

Multiple linear regressions were calculated to predict brain-to-brain synchronization based on the brain-audio synchronization of the Listener and/or the brain-audio synchronization of the Speaker. Different models were implemented to determine the exact sites and bands in which brain-to-brain entrainment is statistically significantly explained by speech-to-brain synchronization (see Statistical Analysis section).

#### Main Full Model

**Figure 3:**
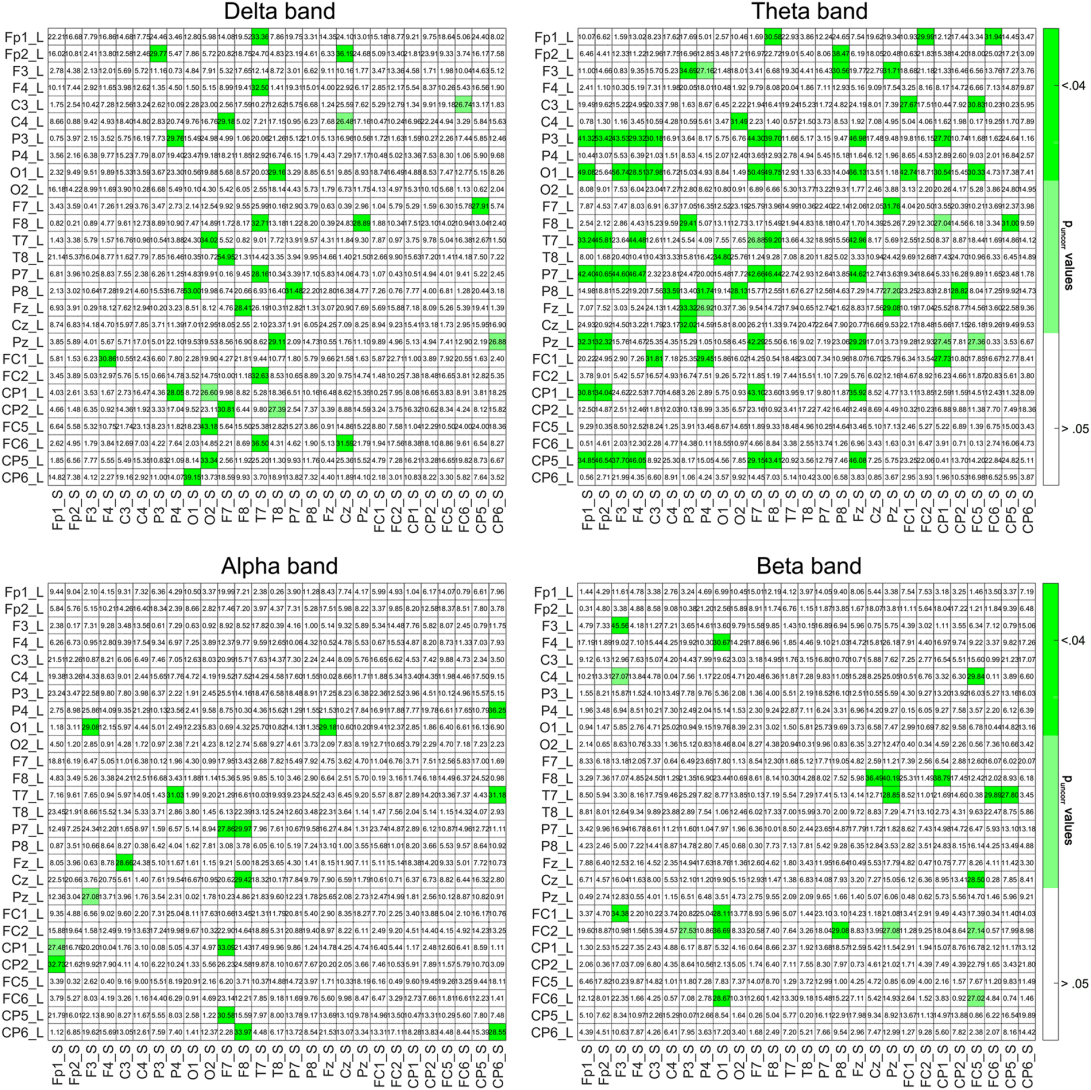
Group-level statistical results from the multiple linear regression modelling analysis (full model) at the different frequency bands. The matrixes contain the coefficient estimates (R^2^ values) for a multilinear regression of the brain-to-brain entrainment response using as predictors the brain entrainment to speech of the listeners and the speakers. Statistically significant percentage of variance explained is indicated by coloring in green the corresponding cells where p < 0.05. Rows represent the electrodes of the Listener and columns represent the electrodes of the Speaker. Both brain-audio synchronizations were significant predictors of the brain-to-brain synchronization in all the analyzed frequency bands, being more generalized in the slow frequencies and mainly in those corresponding to theta.

Results from this model that takes into account all possible effects, including those of listener brain-audio entrainment, speaker brain-audio entrainment, and the interaction effect, are shown in Fig. 3 which contains the coefficients of multiple determination (R^2^) for each channel combination and each frequency band. Cells colored in green indicate that both brain-audio synchronizations (for the Listener and for the Speaker) were significant predictors of the brain-to-brain synchronization. Supplementary Figure 3 contains the corresponding F values (all Fs(2, 28)) associated to the corresponding uncorrected p values from the multiple regression modelling. From the 159 electrode pairs in total that showed a significant effect, only 8 overlapped in at least two bands. This indicates that the contribution of the brain-audio synchronization to the brain-to-brain synchronization is not topographically constrained across bands or driven by the same electrodes. Out of 729 tests in each band, 34 electrode pairs were statistically significant in the case of delta band, 83 in theta, 20 in alpha and 22 in beta band.

#### Partial Models

These more simple models only take into account, separately, the Listener brain-audio entrainment (Model 1) and the Speaker brain-audio entrainment (Model 2) and could be important in explaining results in the brain-to-brain entrainment that go unnoticed in the main full model. Model 1: Results are shown in Supplementary Figure 4. From the 173 electrode pairs in total showing a significant effect, 15 overlapped in at least two bands. For delta, theta, alpha and beta bands, the statistically significant electrode pairs were 46, 57, 17 and 53, respectively. Model 2: Results are shown in Supplementary Figure 5. From the 157 electrode pairs in total showing a significant effect, 13 overlapped in at least two bands. For delta, theta, alpha and beta bands, the statistically significant electrode pairs were 47, 57, 34 and 18, respectively. Partial models explained additional brain-to-brain synchronization mainly in alpha and beta band, thus complementing the results obtained in the main full model.

Interestingly, the larger numbers of electrode combinations that contributed to explaining the brain-to-brain synchronization were in the theta band. Considering the results showing generalized brain-audio envelope synchronization for the Speaker in this same frequency band (which was markedly different from the Listener’s pattern), the possibility cannot unambiguously be ruled out that brain-to-speech entrainment in this band could be mediated by speech articulatory artifacts.

In general, these results suggest the existence of an interbrain coupling process mediated by the Listener’s and/or the Speaker’s brain-to-speech entrainment. In other words, speech is a shared sensory stimulus that explains some of the interbrain coupling data. However, it is important to explore in further detail the brain-to-brain entrainment patterns that cannot be accounted for by the entrainment with speech, given that this would represent a clear instance of seemingly independent interbrain coupling. In the following final analysis, we explored the brain-to-brain synchronization pattern after excluding the electrodes that showed significant brain-audio envelope synchronization for either the Listener or the Speaker (or both) in each of the frequency bands, and also removing the brain-to-brain synchronization predicted by brain-audio synchronization.

### Brain-to-brain synchronization not explained by brain-audio envelope synchronizations

In this final section, the brain-to-brain synchronization effects obtained in each frequency band were explored disregarding (*i*) the electrode combinations for which brain-to-brain synchronization could have been explained by the brain-audio envelope synchronization (as resulting from the multiple linear regression modelling) and (*ii*) the electrode combinations that contain an electrode showing entrainment to speech for the Speaker or the Listener (as resulting from the analysis of the brain-audio envelope synchronization). In other words, channels significantly involved in brain-audio synchronization were fully eliminated from the brain-to-brain synchronization pattern of results. Thus, we report only the brain-to-brain entrainment between speaker and listener that is independent of the auditory processes, since it is not significantly explained by the physical attributes of the auditory stimulus.

**Figure 4:**
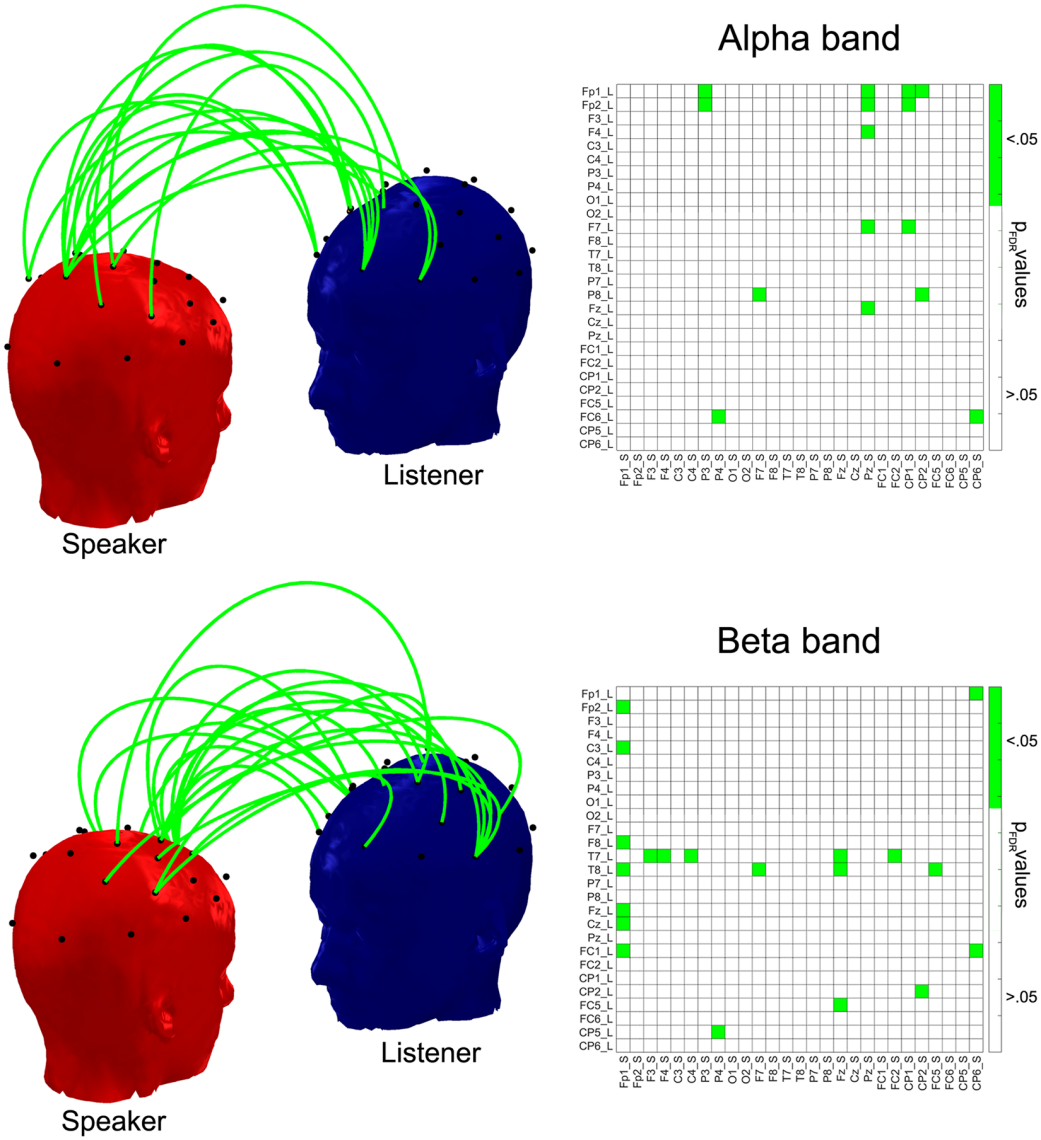
Representation of statistically significant enhanced interbrain synchronization patterns between the listeners and the speakers during oral narratives that are not explained by brain-audio envelope synchronization. Matrices on the right are those reported for group statistics on the interbrain synchronization patterns provided in Fig. 1, after deleting those electrode combinations where brain-to-brain synchronization is explained by the brain-audio envelope synchronization and after deleting electrode combinations that contain at least one electrode showing entrainment to speech. The plots on the left are an alternative representation of the information contained in the matrices. The red head represents the Speaker and the blue head represents the Listener, with the green lines connecting those electrode pairs that show significant enhancement in synchronization. In the alpha band (upper panel) the sites showing interbrain coupling are mainly frontal for the Listener and central for the Speaker. In the beta band (lower panel) the sites are mainly frontal for the Speaker and temporal for the Listener.

With the set of constraints established, no brain-to-brain synchronization effects were expected to survive in the delta and the theta bands, given that in these bands all the channels were involved in the speech-brain synchronization (see Fig. 2 for details). Figure 4 shows the electrode combinations that, independently of sensory entrainment effects, showed enhanced brain-to-brain synchronization during oral narrative interchange at the group level. The right panels represent the matrices from the previous analyses, indicating the electrode pairs whose synchronization pattern cannot be explained by the mediating role of speech (marked in green), and the left panels present the heads plots for the Listener and the Speaker, with green lines connecting the electrode combinations showing a seemingly independent and significant effect. The upper panel shows the interbrain synchronization pattern in the alpha band, which is focal and topographically constrained to the frontal electrodes of the Listener connecting with the central electrodes of the Speaker. For a better view of the topographical distribution of the 15 significant combinations, see Supplementary Video 1. The lower panel shows the pattern in the beta band, which includes 20 combinations involving multiple links between frontal electrodes of the Speaker and temporal electrodes of the Listener. Supplementary Video 2 provides a 3D view of the topographical distribution. Thus, this analysis shows that an important part of the brain-to-brain entrainment pattern found in this study is not the byproduct of a coupling process between the neural oscillatory activity and the shared sensory stimulus (speech), but is mediated by factors other than the physical properties of the utterances.

## Discussion

In the current study we have demonstrated that oral narrative is concomitant with the inter-brain coupling of neural oscillations, a phenomenon that we term ‘brain-to-brain entrainment’. Results here are in line with earlier work^10, 15^ and specifically show an enhanced synchronization between the EEG oscillatory activity of listeners and speakers in delta, theta, alpha and beta frequency bands. The brain synchronization in delta and theta bands was mediated by the speech signal, since entrainment occurred between the neural systems of the senders and the receivers, but also between their brains and the shared message, through entrainment to the audio envelope. Interestingly, some of the synchronization pattern found in the alpha and beta band seems to emerge directly from the mutual interaction of the dyad, without being mediated by the physical properties of speech (i.e., a “pure” instance of brain-to-brain entrainment). These results strongly emphasize the social essence of verbal communication and invite a paradigm shift that does not exclusively focus on the study of individual brain activity in isolation as a proxy for understanding the neural bases of linguistic communication.

The vast majority of studies to date have focused on the relationship between a listener’s neural oscillatory activity and the properties of the perceived speech signal. These studies have provided ample evidence that neural entrainment to speech occurs in the low-frequencies^3, 16–20^. In line with these findings, we found cortical entrainment to the speech envelope mainly in the delta and theta bands. Remarkably, our results demonstrate that the neural oscillators of speakers also align with the verbal signal they themselves produce even including alpha band, which has also been reported as having a role in speech processing^21^. Surprising as it may seem, this is a coherent phenomenon that relies on the fact that language production and comprehension are tightly interwoven^22^, depending to a great extent on similar neural mechanisms^23^. This result is also in line with the idea of corollary discharge, which is a specific instance of an efference copy that allows monitoring and controlling of the interference of one’s own production during ongoing verbal production^24^. We hypothesize that the finding of significant entrainment of speakers to their own speech responds to a form of corollary discharge that permeates the neural system during vocalization.

In addition to concomitant brain entrainment to speech in the delta and theta band, we also found markers of interbrain synchronization at these frequency bands. This novel brain-to-brain entrainment effect may be fully mediated by the speech signal, given that both the speaker and the listener’s neural slow-oscillators also synchronize with the speech signal. In fact, delta-like fluctuation rates correspond to the largest linguistic structures, such as sentences and phrases, whose temporal dynamics tend to map onto these frequency ranges. Theta frequencies, in turn, correspond to the rate at which syllables regularly occur in a continuous speech stream. This finding underscores the role of prosody in language comprehension and highlights the importance of the neural processes underlying the processing of complex linguistic structures in connected speech during interpersonal verbal communication^25, 26^. Hence, we suggest that the interbrain synchronization pattern observed in the slow neural oscillations could be considered a transitive property of a series of brain-to-speech synchronization mechanisms, shared by speakers and listeners, which underlie the encoding of long time-scaled information streams.

It could be tentatively argued that some of the enhanced synchronization patterns found in the present study, especially the speech-to-brain synchronization for the speakers, could be related to jaw movements causing muscle artifacts in the EEG data. However, we can safely conclude that this cannot be the underlying reason for the whole set of brain-to-brain entrainment patterns found, due to several important reasons. First, undetected speech artifacts were also contained to a similar extent in the surrogate data used for the statistical comparisons and therefore no differences should emerge between samples due to this reason. Second, it is important to recall that during the experiment, the two subjects did not move their jaws simultaneously, given the turn-taking strategy followed. And third, the last analysis reported here showed that some of the critical brain-to-brain coupling effects were completely independent of the brain-to-speech entrainment, and consequently not mediated by speech artifacts. Nevertheless, more sophisticated strategies could be used to filter speech artifacts in future studies, such as the use of Common Spatial Filters^27^ or the computation of partial coherence^28^.

Critically, our results also demonstrate the existence of focal interbrain synchronization patterns in the alpha and beta bands. Importantly, part of these data cannot be explained as a function of the presence of a third shared unit acting as the external oscillator modulator (namely, the speech envelope), since the effects remained after taking into account the specific contribution of the brain-to-speech synchronization. Oscillatory activity in alpha frequencies has been typically associated with attention-mediated processing^29–31^, and the role of attention in successful verbal communication is undeniable. That is, attentional resources need to be coordinated among interlocutors not only to understand each other, but also to anticipate forthcoming speech^32^, and to segment the continuous speech signal into meaningful units^33–35^. On the other hand, oscillatory modulations in beta frequencies are supposed to reflect the close relationship between language comprehension and motor functions^36^. It has been shown that the beta rhythm modulation by speech sounds elicits a pattern in the somatosensory cortices of the listener that resembles the activity that subtends actual speech production in the speaker^37^. Furthermore, oscillations in the beta (and theta) band are instrumental in predicting the occurrence of auditory targets^38^. Therefore, it is possible that alpha and beta constitute distinct rhythmic classes that are both involved in top-down processing^39^. Thus, brain-to-brain entrainment in the alpha-beta frequencies could be conceived as part of an emergent property of coupled systems that are mutually dependent due to interactive prediction processes^40^. This claim is reinforced by previous evidence showing increased oscillatory brain activity in the sensorimotor mu rhythm and its alpha-band component, associated with effective social coordination^11, 12, 41^.

The finding that brain-to-brain entrainment during oral narrative emerges (at least in part) from on-line mutual interaction is in good agreement with theoretical accounts postulating that brains work differently in social interactive situations than in isolation^42–47^. Verbal communication should be seen differently from the mere sum of production and perception, and communicative interactions should be conceived as autonomous processes influenced by both the participants and the situation^44^. Therefore, future studies should take advantage of designs that include engaged participants and simultaneous recordings^32^, and should also move beyond the classic event-related designs, to investigating continuous neural encoding^25^. In addition, the current results suggest that the seemingly well-established neural markers of speech production and perception need to be revised, since brain-to-brain coupling also modulates neural activity (and behavior) over and above brain-to-speech coupling, resulting in patterns that do not emerge in isolation^48^.

In summary, the interbrain synchronization patterns between listeners and speakers here evidenced during an exchange of oral narrative support the existence of brain-to-brain entrainment. These results add to a growing body of evidence emphasizing the need for more close-to-natural experimental designs and methodologies that would allow exploration of the complexity of the neural mechanisms underlying verbal communication and social interaction in general.

## Methods

### Participants

The study included data from thirty subjects (16 men, 14 women, mean age: 23.18, SD: 3.64 years, range: 19–31 years). All were right-handed (assessed by an adapted version of the Edinburgh Handedness Inventory^49^), and were native speakers of Spanish. Participants were arranged in 15 pairs or dyads that fulfilled the requisites of: same gender [46], equivalent age (no more than 5 years of difference), and not knowing each other prior to the experiment. We had three a-priori exclusion criteria (*i*) technical issues during data acquisition such as saturation of the amplifiers, high electrode impedance and electrode detachment, (*ii*) large artifacts due to laughing, sneezing, coughing, yawning, body movements or tics, and (*iii*) participant discomfort with the experimental set-up. Data from one additional dyad pair was excluded due to technical issues during recording. All participants had adequate hearing, (corrected to) normal vision and had no known neurological/psychiatric disorders or current drug abuse. Participants were recruited from the participant pool of the Basque Center on Cognition, Brain and Language (BCBL). Individual written informed consent was obtained from all participants prior to the experimental session, which included consent for their de-identified data to be freely available for further research. All participants received monetary compensation (€15/h) for the EEG session which lasted approximately 2 hours, plus an additional €5 for a post-scan questionnaire used to evaluate each participant’s attention during the experimental procedure. The study was conducted in accordance with the Declaration of Helsinki (1964) and approved by the BCBL Ethics Committee.

### Experimental setting

**Figure 5:**
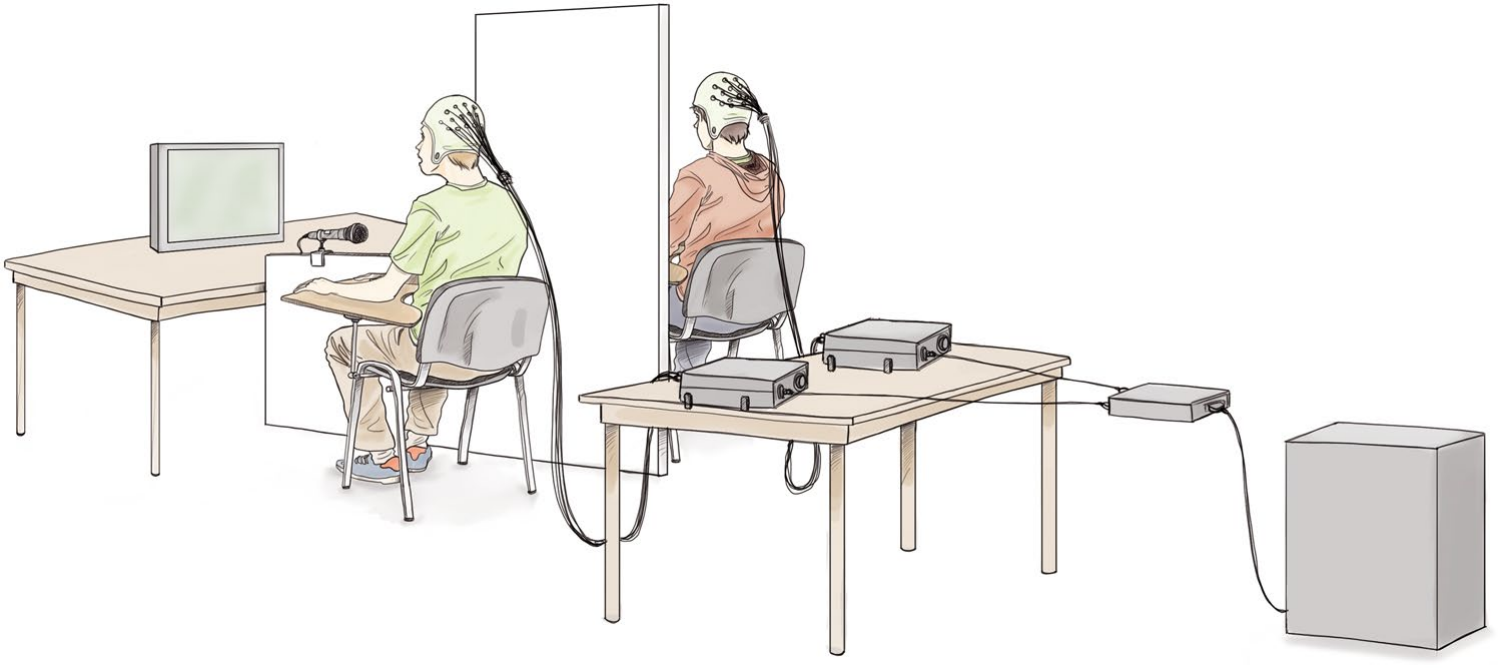
Experimental set-up.

Pairs of participants were introduced to each other and the experimental procedure, and were engaged in small-talk with each other and the experimenters during the setting up of the EEG cap and electrodes. The experiment was conducted in a soundproof chamber. Figure 5 shows a depiction of the experimental set-up. Participants were seated side by side, with a board placed between them that prevented them from seeing each other. This was done in order to avoid possible entrainment resulting from embodied interaction, such as those due to non-verbal (visual) communication through glances, frowns, gesticulations and posture^15^, and more importantly, due to lip movements^28^. A microphone was attached to the board separating the dyad pair in order to record the verbal production of both participants. A computer screen facing participants was placed at a distance of approximately 1.5 meters, centered, and was equally visible to the two participants. Detailed instructions and explanations about the experiment were provided. Participants were asked to (*i*) be relaxed and still, avoiding body movements (e.g. gesticulation, nodding); (*ii*) pay careful attention to the content of the partner’s verbal production; and (*iii*) speak continuously and without much pausing, vocalize clearly and avoid whispering and the use of interjections when cued to speak. Presentation of all instructions and stimuli was controlled by a custom-written program created and compiled with Experiment Builder© software (SR-Research, Ontario, Canada) that was run on a PC. The program also recorded the audio files and sent the triggers to the PC used for the EEG recordings. All participants were informed that a short evaluation about the content of the partner’s verbal productions would take place at the end of the experiment and that they would receive a small monetary reward based on successful performance in the evaluation (24 correct responses out of the 30 questions).

### Task

Pairs of subjects listened to each other’s semi-structured oral narratives without interpersonal visual contact. Specifically, participants were required to alternate the roles of speaker and listener while interchanging verbal information about their preferences and opinions about 5 different common topics: sports, movies, animals, music and travel. Depending on their assigned role, participants were asked to either speak as freely and naturally as possible about the corresponding topic, or to listen paying careful attention to the other’s speech. Each topic was accompanied by a series of 6 questions that were presented on the screen as a guide. Participants were asked to answer each question as fully as possible, since these questions would be used in the evaluation at the end of the experiment, to assess the degree of attention paid to the partner. Each trial lasted for 180 seconds. For the first 90 seconds, one participant was assigned the role as speaker, and the other as listener, and the roles were reversed for the second half of the trial. Interval between trials and within trials (exchange of roles) had a length of 15 seconds. These intervals minimize the potential synchronization of respiratory and cardiac activity that may occur in smooth turn-taking^50^. Together with the topic and the guide, a cue (blue arrow) indicating the participant of the dyad who had to speak was presented on the screen. Participants were also presented in a corner of the screen with information about the remaining time of each 90 seconds turn, by a countdown clock that showed the time at irregular intervals. A trial in which both participants had to remain silent was also included as a baseline (see *EEG preprocessing* section). The presentation order of topics and the silent block, and the initial role order within a trial were randomized across dyads. One additional trial (topic: food) was used for practice purposes at the beginning of the experiment and was not included in the analysis. Therefore, the experiment consisted of 5 listening events (Listening condition), 5 speaking events (Speaking condition) and one silent event (Resting condition) for each participant in each dyad. After finishing the EEG recording session, participants were asked to complete a paper and pencil evaluation concerning the information their partner provided during the experimental session (which had been cued by the guided questions). Each participant then scored their partner’s evaluation (1 point for each correct response).

### EEG hyperscanning recordings

Electrophysiological signals of each subject were acquired from 32-channels. Each subject was connected through an individual Electrode Input Box to one BrainAmp Standard amplifier (Brain Products GmbH), allowing for individual Reference and Ground electrodes. The two BrainAmps (32-channles) were synchronized by the same USB 2 Adapter (BUA). Data was recorded using custom workspaces in BrainVision Recorder. EEG signals were acquired using elastic caps (EasyCap) mounted with 27 scalp Ag/AgCl electrodes placed according to the International 10–20 system, and included: Fp1/Fp2, F3/F4, F7/F8, FC1/FC2, FC5/FC6, C3/C4, T7/T8, CP1/CP2, CP5/CP6, P3/P4, P7/P8, O1/O2, Fz, Cz, and Pz. Ground electrodes were placed at AFz location. Additional clip-on electrodes were located on the earlobes, with left one used as online reference. Two electrooculography (EOG) electrodes were placed at the external ocular canti (to monitor horizontal eye movements), and two electromyography (EMG) electrodes were placed at the left orbicularis oris superior and left orbicularis oris inferior (halfway between the center and the corner of the mouth). Impedance measurements were checked by two individual 32-channels workspaces corresponding to each participant/amplifier. Inter-electrode impedances were set below 5 kΩ at the beginning of the experiment. Data were acquired at a sampling rate of 250 Hz. EEG markers were time-locked to the beginning of each listener/speaker turn.

### EEG preprocessing

The data were analyzed using EEGLAB toolbox^51^ and custom programs that were run in MATLAB (version 2014b, The MathWorks Inc.). The recorded signal of each individual participant was re-referenced off-line to the right-earlobe electrode, and a high-pass filter (FIR) at 1 Hz was applied. Bad channels (identified with the PREP toolbox^52^ and through visual inspection of the power spectra) were removed from the dataset. A low-pass filter at 20 Hz was applied in order to reduce the presence of muscular artifacts^53^, after which an Independent Component Analysis (ICA, runica method, EOG channels included) was performed to identify additional artifacts. Components identified as mainly containing ocular movements (i.e., blinks, saccades), heartbeat and muscle artifacts (by examining the components’ topographies, frequency spectra and time courses) were removed (up to 4 components; mean: 3). Components that were highly correlated with the EMG signals (p < 0.0001) were also removed. Bad channels were then interpolated. Finally, the 90-second epochs of interest were extracted from the signal of each participant (11 epochs; 5 production events, 5 listening events, 1 silent event).

### Brain-to-brain synchronization (brain-to-brain entrainment)

The phase synchrony between the Speaker and Listener electrode pair signals was calculated by using the phase locking value (PLV) measure^54^. The measurements were performed on a trial-basis, using the 90-second epochs. Specifically, PLV from all possible combinations of electrode pair signals (n = 729) corresponding to the two different but concurrent EEG recordings in the dyad was estimated. This measure reflects the mean phase coherence of an angular distribution. A PLV variant that involves averaging the instantaneous phase differences over time within a single trial (*n*) was used. This variant is expressed in equation (1), where *T* is the number of time points, ϕ_(*t, n*)_ is the phase of trial at time *t*, in channel ϕ, and ψ_(*t, n*)_ in channel ψ. Instantaneous phase at each time point in each time series was estimated from the Hilbert Transform at 4 frequency bands: delta (1–3 Hz), theta (4–8 Hz), alpha (9–12 Hz) and beta (15–20 Hz). The PLV varies between 0 (random phase) to 1 (perfect phase-locking). This form of the PLV is essentially a measure of the intra-trial consistency of the phase difference between channels and it has been previously used in EEG hyperscanning studies^55^. The outputs are matrices with dimensions 27 × 27 × 4 × 6 (i.e. Listener’s channels × Speaker’s channels × frequency bands × blocks corresponding to the five topics plus the resting condition). Supplementary Figure 4 contains these outputs collapsed across topics and subjects.

1 $$PL{V}_{t}=\frac{1}{T}|\sum _{n=1}^{T}{e}^{i({\varphi }_{(t,n)}-{\psi }_{(t,n)})}|$$

PLVs obtained in the resting condition were subtracted to those obtained in each of the topics^15^. This was done to avoid non-specific kinds of engagement that may result from physical cohabitation, and thus allows us to confidently claim that any synchronization effects obtained have been solely driven by the linguistic interaction. Finally, data were collapsed across topics. This resulted in estimations of synchronization between the 27 channels of the Speaker and the 27 channels of the Listener in each frequency band. As the Speaker-Listener situation is twofold inside the dyad, the final sample is composed of 30 cases.

### Brain-audio envelope synchronization (brain entrainment to speech)

The brain-to-speech synchronization was estimated (*i*) between the Listener’s EEG signals and the Speaker’s amplitude envelope, and (*ii*) between the Speaker’s EEG signals and their own amplitude envelope. First, we computed the amplitude envelope for each audio waveform, extracted through the computation of a power spectrogram and resampled to 250 Hz. Second, the phase synchronization between the amplitude envelope and the EEG neural oscillation (in each channel) was directly quantified by using the same PLV measure and similar procedure as described for the brain-to-brain case (Supplementary Figure 5 contains these outputs collapsed across topics and subjects). Third, intra-brain PLV between all sensor pairs was estimated. Fourth, intra-brain PLV was averaged across sensor pairs and the resulted values were extracted from the brain-amplitude envelope PLV at each frequency^18^. The obtained brain-amplitude envelope PLV was finally collapsed across topics. This synchronization estimation resulted in the following output for each participant and role: 27 channels × 4 frequency-bands.

### Surrogate datasets

Brain-to-brain: surrogate data were created with signals taken from different trials but preserving within the dyad each participant’s role (Speaker or Listener). For example, the EEG signal of participant A listening about ‘sports’, was paired with the EEG signal for participant B speaking about ‘movies’. By shuffling the trials, 20 possible combinations were obtained.

Brain-to-speech: similarly to above, the amplitude envelopes were paired with the EEG signals recorded from other trials. For example, subject A’s speech amplitude envelope for ‘sports’ was paired with the EEG of subjects A/B listening/speaking about ‘movies’).

It was assumed that no significant increases in synchronization would be found for surrogate datasets since the signal segments were not synchronously taken. Importantly, given that the surrogate datasets were created with non-time corresponding data that exactly reproduces the roles of listener and speaker from the real dataset, the same physical attributes (e.g. pitch, intonation and breathing patterns) and data distribution were maintained. The same data treatment procedure for the analysis of synchronization described for real data was also applied to the surrogate datasets.

### Statistical Analysis

In order to assess statistical differences in the phase coupling measurements between the real and the surrogate dataset (both in the brain-to-brain and in the brain-audio envelope analyses), a nonparametric bootstrap-based t-test method from EEGLAB’s Resampling Statistical Toolkit was used. The bootstrap test is a distribution-free test and does not require any assumptions about the correlation structure of the data. The number of random sampling was set to 10000. An FDR correction^56^ for multiple comparisons (q = 0.05) was applied for the p values obtained (all frequency bands jointly). Statistically significant increases (compared to the surrogate data) are interpreted as showing neural entrainment to speech for the case of brain-envelope synchronization data, and as showing interbrain synchronization for the case of the brain-to-brain synchronization data.

In order to directly statistically test if the brain-to-brain entrainment was mediated by speech-to-brain entrainment, a multiple linear regression modelling was used. Specifically, we obtained the coefficient estimates (percentage of variance explained and associated p values) of a multilinear regression of the brain-to-brain entrainment results (at each channel pair) using as predictors the corresponding results from the brain entrainment to speech analysis (corresponding channels) for each of the 30 observations. This procedure was performed separately for each frequency band. The main “full” model takes into account all possible effects, including those of listener brain-audio entrainment, speaker brain-audio entrainment, and the interaction effect. It is expressed in the typical linear regression modelling equation (2) where: *Y* is the brain-to-brain entrainment for channel ‘A’ of the Listener and channel ‘B’ of the Speaker, *x*_1_ is the Listener’s brain entrainment to speech at channel ‘A’, *x*_2_ is the Speaker’s brain entrainment to speech at channel ‘B’ and *x*_1_ *x*_2_ their interaction.

2 $${Y}={\beta }_{0}+{\beta }_{1}{x}_{1}+{\beta }_{2}{x}_{2}+{\beta }_{12}{x}_{1}{x}_{2}+\in $$

In addition, two more parsimonious models were also used to apprehend any effect that could go unnoticed in the full model. In those models only listener brain-audio (*Y = β*_0_ + *β*_1_ *x*_1_ + *ϵ*) and speaker brain-audio entrainment (*Y = β*_0_ + *β*_1_ *x*_2_ + *ϵ*) were used as predictors. Statistically significant results in any model were interpreted as showing the specific contribution of speech-brain entrainment to brain-brain entrainment.

### Code availability

All code can be requested from the corresponding author.

### Data availability

The datasets generated during and/or analyzed during the current study are available from the corresponding author on reasonable request.

## Electronic supplementary material

Supplementary Information

Supplementary Video 1

Supplementary Video 2
