# Supplementary material for: Brain-to-brain entrainment: EEG interbrain synchronization while speaking and listening
Source: Sci Rep. 2017 Jun 23;7:4190. doi: 10.1038/s41598-017-04464-4 (PMC5482847; doi:10.1038/s41598-017-04464-4)
Supplement: Supplementary file 1 — Supplementary Information [file 41598_2017_4464_MOESM1_ESM.pdf]

Brain-to-brain entrainment: EEG interbrain synchronization while speaking and listening.

Authors: Alejandro Pérez[*], Manuel Carreiras & Jon Andoni Duñabeitia.

[*]Corresponding author

t values from the brain-to-brain synchronization analysis

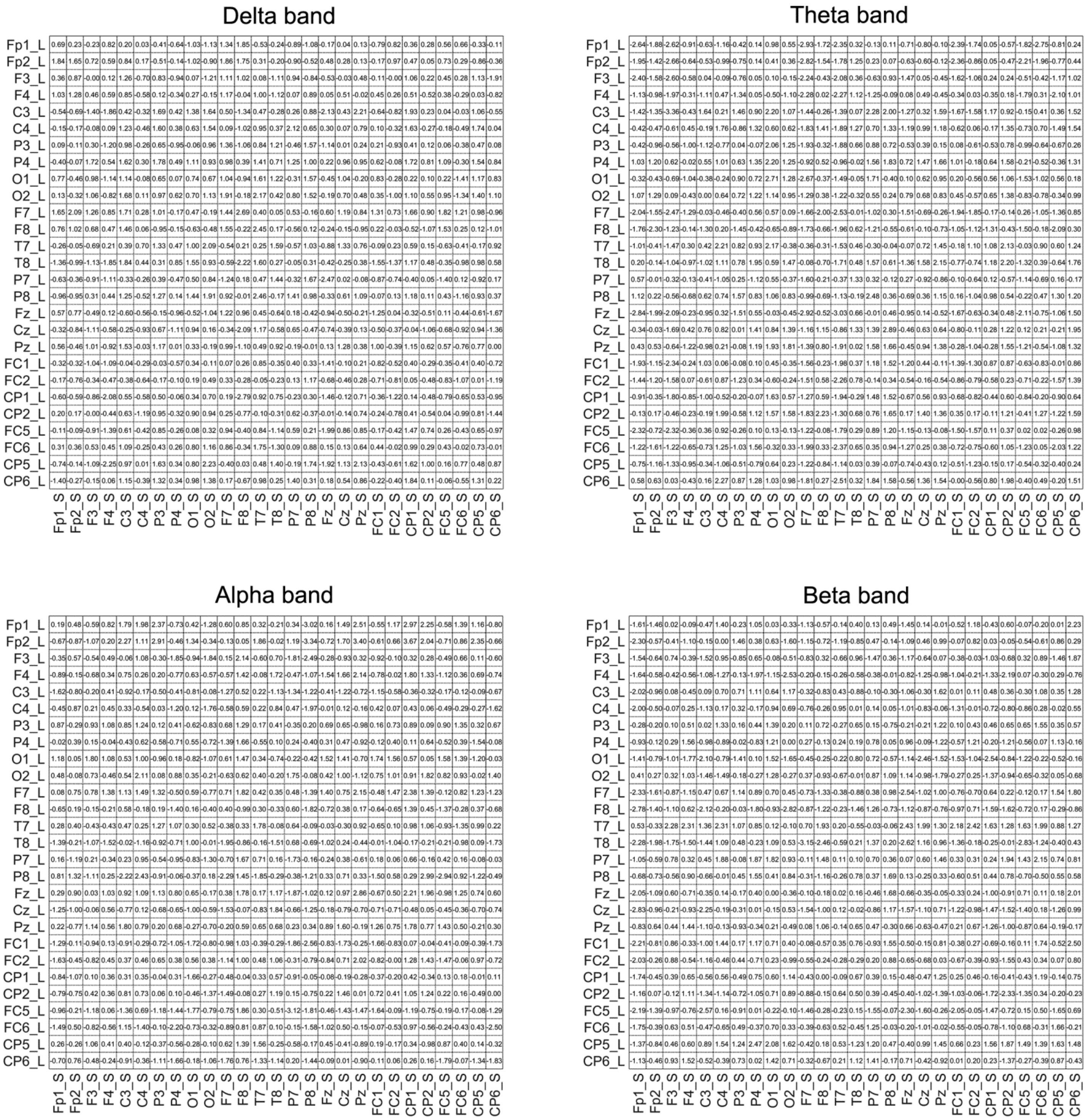

Supplementary Figure 1: Group-level statistical results from the brain-to-brain synchronization analysis at the different frequency bands. The matrices contain the t values from nonparametric bootstrap-based t-test method.

t values from the brain-audio synchronization analysis

	Listener-Delta	Speaker-Delta	Listener-Theta	Speaker-Theta	Listener-Alpha	Speaker-Alpha	Listener-Beta	Speaker-Beta
Fp1	2.92	7.06	1.71	5.33	0.05	1.32	-0.59	0.55
Fp2	3.94	6.38	1.33	5.85	0.03	1.73	0.72	-0.43
F3	3.28	6.33	2.15	5.30	0.67	1.57	0.48	0.79
F4	3.15	6.08	2.52	6.09	1.52	0.86	0.24	0.88
C3	3.79	5.26	-0.54	3.64	0.41	1.73	-0.20	1.69
C4	3.05	4.31	0.94	5.55	0.00	2.08	0.38	0.32
P3	2.90	5.24	-1.37	3.10	-1.16	1.30	-0.18	-0.23
P4	2.38	4.91	-0.94	4.12	0.62	1.12	0.09	-0.26
O1	2.36	6.55	-1.33	3.47	0.42	1.77	-2.26	0.86
O2	1.66	7.06	0.99	3.75	0.51	0.43	0.44	1.50
F7	2.59	6.04	0.50	4.79	-0.03	1.08	1.74	0.86
F8	3.52	5.44	1.46	6.10	1.18	2.34	0.85	0.90
T7	3.15	5.32	0.22	5.82	-0.48	2.22	0.99	1.48
T8	3.68	6.29	1.26	7.13	0.53	1.28	0.70	0.33
P7	3.61	4.94	-1.51	5.78	-0.76	2.37	-2.01	-0.83
P8	2.01	6.10	0.79	5.69	0.81	1.86	0.19	1.00
Fz	3.08	7.39	2.20	5.42	1.26	2.14	-0.32	0.60
Cz	3.16	4.78	0.42	5.21	1.32	3.04	-0.41	2.21
Pz	2.04	4.87	-1.13	3.24	0.74	1.00	-0.82	0.94
FC1	3.48	5.93	1.35	4.61	1.29	2.86	-0.31	1.40
FC2	3.14	5.17	1.85	5.68	1.20	1.95	0.77	0.56
CP1	2.43	4.30	-0.55	3.76	-0.49	1.31	-1.12	0.78
CP2	1.87	3.84	-0.78	4.45	0.01	1.07	-0.80	0.07
FC5	3.77	7.42	1.94	4.74	0.65	1.74	0.49	1.28
FC6	3.76	5.97	2.15	6.19	-0.05	2.05	1.12	0.08
CP5	3.00	5.52	-1.90	3.88	-0.28	2.01	-0.63	0.33
CP6	2.64	4.50	0.04	4.90	-0.17	1.16	-1.31	0.46

Supplementary Figure 2: Group-level statistical results from the brain-audio envelope synchronization analysis of the Listener and the Speaker at the different frequency bands. The matrix contains the t values from nonparametric bootstrap-based t-test method.

F values from the multiple linear regression modelling

Delta band

	Fp1_S	Fp2_S	F3_S	F4_S	C3_S	C4_S	P3_S	P4_S	O1_S	O2_S	F7_S	F8_S	T7_S	T8_S	P7_S	P8_S	Fz_S	Cz_S	Pz_S	FC1_S	FC2_S	CP1_S	CP2_S	FC5_S	FC6_S	CP5_S	CP6_S
Fp1_L	2.47	1.74	0.73	1.76	1.49	1.87	2.81	0.31	1.27	0.55	1.42	2.10	4.34	0.74	2.13	0.30	1.45	2.75	1.30	1.55	2.00	0.88	0.94	1.99	0.46	2.80	0.76
Fp2_L	1.65	1.05	0.21	1.39	1.25	1.23	3.67	0.50	0.74	0.53	2.28	2.00	2.84	0.44	2.62	0.42	0.59	4.92	2.84	0.46	1.34	2.42	2.72	0.89	0.34	1.67	0.71
F3_L	0.25	0.40	0.19	1.18	0.52	0.53	1.09	0.06	0.44	0.74	0.49	1.86	1.20	0.83	0.27	0.61	0.87	0.98	0.16	0.31	1.34	0.42	0.15	0.17	0.97	1.49	0.47
F4_L	0.97	0.70	0.26	1.14	0.36	1.25	0.12	0.41	0.13	0.47	0.86	2.09	4.17	0.12	2.07	1.53	0.36	2.58	0.57	0.25	1.20	0.51	0.79	0.99	0.50	1.72	0.17
C3_L	0.15	0.23	1.01	0.68	1.25	1.32	0.23	0.97	0.20	2.59	0.23	1.85	0.99	1.25	1.62	0.62	0.11	2.98	0.72	0.48	1.27	0.12	0.95	2.06	3.16	1.31	0.16
C4_L	0.82	0.08	0.90	0.45	1.95	1.51	0.25	2.27	0.94	1.74	3.57	0.46	0.67	1.79	0.08	0.58	0.72	3.12	1.79	1.01	0.99	1.77	2.48	0.45	0.30	0.54	1.61
P3_L	0.07	0.36	0.19	0.32	0.53	1.67	0.73	3.67	1.59	2.89	0.45	0.09	2.17	2.34	1.54	2.31	0.47	1.77	1.02	1.15	1.14	1.14	0.99	0.20	1.83	0.54	1.23
P4_L	0.32	0.19	0.59	0.94	1.56	0.73	0.76	2.09	2.66	2.06	1.93	1.16	1.29	1.74	0.57	0.16	0.40	0.68	1.80	1.01	0.46	1.34	0.71	0.78	0.09	0.54	0.93
O1_L	0.21	0.91	0.91	0.95	1.57	1.36	0.33	2.63	1.02	2.15	0.52	0.81	2.17	3.57	0.29	0.84	0.60	0.95	0.85	2.00	1.71	1.52	0.81	0.70	1.27	0.47	0.78
O2_L	1.67	1.44	0.86	1.15	0.35	0.99	0.62	0.50	0.97	0.39	0.50	0.56	0.23	1.92	0.40	0.53	0.16	0.63	1.15	0.37	0.45	1.57	0.97	0.52	0.10	0.05	0.18
F7_L	0.31	0.32	0.04	0.68	1.10	0.34	0.31	0.24	0.19	1.24	0.95	0.91	3.04	0.98	1.28	0.34	0.05	0.03	0.26	0.09	0.53	0.48	0.14	0.58	1.62	3.36	0.53
F8_L	0.07	0.02	0.08	0.43	0.92	1.26	0.85	1.06	0.70	1.52	1.15	0.77	4.21	1.32	1.10	0.77	0.03	2.86	3.52	0.17	1.00	1.84	2.60	1.41	1.07	1.30	1.23
T7_L	0.13	0.30	0.53	0.14	1.74	1.07	1.02	1.40	2.78	4.47	0.51	0.07	0.86	0.73	1.40	0.92	0.39	1.16	0.89	0.74	0.08	0.34	0.94	0.46	1.70	1.26	1.13
T8_L	2.32	1.57	1.66	0.83	1.14	0.73	0.74	1.71	1.00	1.04	10.57	2.35	1.46	0.30	0.36	0.96	1.49	0.12	2.37	1.26	0.95	1.61	1.80	1.12	1.43	0.70	0.67
P7_L	0.63	0.36	0.99	0.84	0.71	0.21	0.58	1.10	1.51	2.15	0.01	0.90	3.40	1.00	0.30	1.79	0.54	1.42	0.43	0.09	0.04	1.02	0.45	0.36	0.90	0.48	0.22
P8_L	0.19	0.27	1.03	1.81	2.06	0.42	1.59	1.75	9.77	2.16	0.63	2.26	0.65	1.70	3.98	2.47	1.27	1.70	0.43	0.68	0.07	0.73	0.36	0.63	0.11	2.23	0.28
Fz_L	0.65	0.35	0.03	1.92	0.71	1.29	0.98	0.29	0.81	0.77	0.43	3.44	3.08	1.00	1.27	1.10	0.27	2.29	0.72	0.52	1.64	0.67	0.76	0.48	0.49	2.09	0.12
Cz_L	0.83	0.63	1.43	0.43	1.65	0.74	0.33	1.11	1.78	1.29	1.91	0.23	0.19	2.64	0.17	0.56	2.78	0.66	0.78	0.85	0.88	1.58	1.32	0.15	0.26	1.64	1.76
Pz_L	0.35	0.54	0.36	0.52	0.52	1.78	0.46	2.46	2.10	2.10	0.81	1.76	0.82	3.56	0.19	1.50	1.02	0.15	1.08	0.95	0.45	0.47	0.45	0.69	1.28	0.19	3.19
FC1_L	0.53	0.13	0.58	3.87	1.02	1.23	0.61	0.73	0.20	2.15	0.39	2.42	0.90	1.05	0.16	0.53	0.93	2.39	0.14	0.54	2.55	1.07	0.35	0.74	2.24	0.14	0.21
FC2_L	0.31	0.35	0.46	1.29	0.53	0.47	0.06	1.50	0.32	1.50	0.96	1.09	4.20	0.81	1.03	0.85	0.29	0.94	1.50	0.13	0.99	0.69	1.96	1.73	0.34	1.27	0.49
CP1_L	0.36	0.23	0.32	0.15	0.24	1.71	0.40	3.38	0.83	3.14	0.96	0.84	0.48	1.95	0.60	0.98	1.71	0.82	1.57	0.99	0.75	0.76	1.73	0.34	0.85	0.34	1.93
CP2_L	0.42	0.13	0.59	0.08	1.45	1.17	0.30	1.78	0.91	2.61	3.86	0.60	0.94	3.27	0.23	0.69	0.30	0.84	1.48	0.29	0.34	1.69	1.03	0.79	0.38	0.77	1.63
FC5_L	0.62	0.51	0.49	1.04	2.41	2.61	0.78	1.16	1.93	6.59	0.52	1.59	2.95	1.27	1.56	0.35	0.08	1.51	1.56	0.84	0.71	1.08	1.21	1.02	2.74	2.74	1.95
FC6_L	0.23	0.45	0.16	0.35	1.26	0.66	0.38	0.72	0.18	1.51	0.20	0.82	4.98	0.39	0.42	0.17	0.47	4.00	2.41	0.17	1.85	1.95	1.92	0.84	0.92	0.61	0.78
CP5_L	0.16	0.61	0.73	0.51	0.50	1.57	1.05	2.32	0.77	4.33	0.23	1.17	2.92	1.10	0.96	1.16	0.04	2.94	1.59	0.44	0.68	1.68	1.33	1.73	2.14	0.83	0.62
CP6_L	1.51	0.69	0.37	0.20	2.05	0.26	1.07	1.42	5.58	1.38	1.98	0.96	0.33	2.02	1.39	0.68	0.40	1.17	1.42	0.19	0.27	1.05	0.78	0.30	0.54	0.72	0.32

Theta band

	Fp1_S	Fp2_S	F3_S	F4_S	C3_S	C4_S	P3_S	P4_S	O1_S	O2_S	F7_S	F8_S	T7_S	T8_S	P7_S	P8_S	Fz_S	Cz_S	Pz_S	FC1_S	FC2_S	CP1_S	CP2_S	FC5_S	FC6_S	CP5_S	CP6_S
Fp1_L	0.97	0.61	0.14	1.30	0.78	1.85	1.86	0.46	0.23	1.01	0.15	3.82	2.58	0.35	1.21	2.83	0.71	2.12	2.08	1.06	3.71	1.19	1.83	0.30	4.07	1.46	0.31
Fp2_L	0.60	0.40	1.22	1.10	1.29	1.87	1.77	1.28	0.31	1.80	1.77	2.55	2.03	0.49	0.76	5.42	0.57	1.91	2.23	1.03	2.42	1.57	1.43	1.90	2.89	1.80	0.28
F3_L	1.07	1.49	0.07	0.89	1.61	0.48	4.60	3.23	2.37	1.90	0.31	0.62	2.07	0.40	1.70	3.81	2.14	2.56	4.03	1.99	2.33	2.35	1.71	0.61	1.38	1.81	0.34
F4_L	0.21	0.10	0.99	0.47	0.68	1.18	2.17	1.90	1.01	0.17	0.94	0.76	2.17	0.16	0.66	1.29	0.47	0.87	1.84	0.29	0.77	0.77	1.50	0.62	0.67	1.51	0.95
C3_L	2.10	2.11	1.56	2.88	2.21	0.75	0.14	0.82	0.60	0.20	2.44	1.70	2.07	1.56	1.15	0.44	2.77	0.76	0.69	3.32	1.84	1.01	0.75	3.86	0.99	0.99	0.55
C4_L	0.07	0.11	0.10	0.31	1.03	0.39	1.03	0.51	0.31	3.98	0.20	0.12	0.05	2.37	0.34	0.81	0.17	0.66	0.45	0.46	0.37	1.14	0.17	0.01	2.07	1.15	0.74
P3_L	6.10	9.94	6.68	3.60	3.75	1.76	0.33	0.77	0.53	0.63	6.89	5.70	1.14	0.47	0.28	0.91	7.68	1.84	0.91	2.14	1.67	3.32	1.04	1.15	1.14	2.83	0.10
P4_L	1.01	1.30	0.51	0.59	2.31	0.13	0.81	0.38	0.18	1.23	1.37	1.29	0.25	0.45	0.50	1.55	1.14	0.56	0.17	0.82	0.41	1.28	0.23	0.86	0.18	1.75	0.23
O1_L	8.36	2.99	11.37	3.46	5.31	1.74	1.53	0.45	0.84	0.13	8.84	8.58	1.29	0.12	0.59	1.42	16.92	1.35	0.10	6.47	1.99	3.81	1.58	3.77	0.43	1.82	0.69
O2_L	0.76	0.86	0.71	0.56	2.59	1.81	1.27	0.82	1.05	0.08	0.64	0.62	0.48	1.38	1.32	2.07	0.16	0.22	0.35	0.28	0.19	2.20	0.38	0.48	0.35	2.86	1.52
F7_L	0.74	0.41	0.70	0.76	0.64	0.59	1.78	1.69	1.24	2.62	3.01	1.41	1.53	1.00	2.50	2.46	1.19	1.19	4.03	0.36	2.23	1.36	2.22	0.99	1.37	1.22	0.36
F8_L	0.23	0.19	0.26	0.40	1.56	0.92	3.61	0.46	1.31	1.26	0.28	1.59	2.44	0.44	1.93	1.01	0.15	1.46	2.93	0.68	1.22	3.21	1.48	0.57	0.30	3.89	0.92
T7_L	4.32	7.33	2.68	6.94	1.25	1.10	0.51	0.37	0.71	0.72	3.19	12.58	1.37	0.39	2.03	1.60	6.53	0.77	0.52	1.24	1.24	0.79	0.84	1.96	1.15	1.51	1.43
T8_L	0.75	0.15	2.22	1.01	1.01	1.33	1.63	1.70	4.63	3.01	1.10	0.89	0.66	0.77	1.16	0.46	0.30	1.06	2.80	0.93	1.26	1.83	2.84	1.07	0.59	0.60	1.52
P7_L	6.38	5.93	6.98	7.52	0.21	2.71	2.81	2.17	1.59	1.87	6.45	7.51	2.55	0.26	1.25	1.39	6.98	1.27	1.37	2.08	1.99	0.49	1.69	0.95	1.14	2.66	0.16
P8_L	1.53	2.01	1.56	2.06	1.85	4.38	1.34	4.03	2.05	3.39	1.62	1.24	1.14	0.58	1.24	1.48	0.68	1.50	3.24	2.63	2.71	1.27	3.51	0.76	1.81	2.16	1.50
Fz_L	0.66	0.70	0.27	0.48	2.76	1.12	4.33	3.19	1.00	0.69	0.91	1.50	1.89	0.06	1.27	2.39	0.84	1.85	3.55	0.98	1.78	2.97	2.00	1.48	1.36	2.53	0.89
Cz_L	2.88	2.29	1.47	1.32	1.16	2.61	4.08	1.48	1.63	0.75	3.08	1.11	0.94	2.23	2.54	0.74	2.27	1.73	0.91	2.47	1.96	1.61	1.79	3.07	2.07	2.10	0.91
Pz_L	4.14	4.14	1.62	1.49	2.94	0.39	1.56	0.09	1.03	0.60	6.35	2.97	0.57	0.86	0.67	2.60	3.59	1.78	0.34	2.07	1.29	3.28	0.73	3.26	0.03	0.32	0.62
FC1_L	2.20	2.88	0.26	0.68	4.04	0.67	2.94	3.62	1.63	1.65	1.44	0.05	1.96	2.59	0.69	1.07	1.91	1.74	3.01	0.59	1.36	3.33	1.05	1.88	1.73	1.27	0.80
FC2_L	0.34	0.86	0.50	0.51	1.72	0.45	1.74	0.70	0.88	0.53	1.17	0.10	0.70	1.59	0.10	0.68	0.53	0.56	1.20	1.49	0.85	1.68	0.42	1.17	2.28	0.51	0.34
CP1_L	3.86	4.47	2.83	2.52	1.86	1.49	0.29	0.26	0.53	0.08	6.56	2.68	1.40	0.88	0.94	1.17	4.86	0.81	0.43	1.31	1.39	1.25	1.14	1.47	1.23	1.11	0.76
CP2_L	1.24	1.51	0.22	1.23	1.16	1.18	0.98	0.86	0.30	0.61	2.61	1.06	0.31	1.80	0.69	1.71	1.24	0.83	0.41	1.00	0.99	1.76	0.95	1.11	0.72	0.70	1.95
FC5_L	0.89	1.00	0.81	1.24	1.93	0.11	0.35	1.35	0.82	1.49	1.17	0.89	1.96	0.45	0.99	1.49	1.35	0.47	1.79	0.22	0.48	0.48	0.64	0.12	0.63	1.53	0.31
FC6_L	0.04	0.42	0.18	1.22	0.20	0.43	1.46	0.01	1.97	1.07	0.42	0.84	0.30	0.23	1.38	0.11	0.65	0.31	0.14	0.03	0.60	0.35	0.06	0.01	0.24	1.66	0.43
CP5_L	4.64	7.55	5.24	7.40	0.85	2.94	0.77	1.54	0.41	0.74	3.57	6.65	2.29	0.32	1.27	0.70	7.41	0.68	0.53	2.63	2.45	0.04	1.38	1.43	2.57	2.86	0.47
CP6_L	0.05	0.24	2.44	0.39	0.61	0.85	0.09	0.38	0.32	0.95	1.46	0.66	0.47	1.01	0.27	0.61	0.35	0.07	0.33	0.26	0.35	0.17	1.02	1.77	1.71	0.55	0.35

Alpha band

	Fp1_S	Fp2_S	F3_S	F4_S	C3_S	C4_S	P3_S	P4_S	O1_S	O2_S	F7_S	F8_S	T7_S	T8_S	P7_S	P8_S	Fz_S	Cz_S	Pz_S	FC1_S	FC2_S	CP1_S	CP2_S	FC5_S	FC6_S	CP5_S	CP6_S
Fp1_L	0.90	0.86	0.19	0.37	0.89	0.68	0.59	0.39	1.02	0.30	2.17	0.67	0.21	0.02	0.35	1.10	0.80	0.73	0.38	0.55	0.45	0.09	0.57	1.42	0.07	0.61	0.75
Fp2_L	0.54	0.53	0.47	0.99	1.44	1.70	1.95	0.21	0.82	0.25	1.83	0.67	0.36	0.40	0.68	0.48	1.84	0.55	0.78	0.30	0.95	0.77	1.25	1.95	0.81	0.73	0.34
F3_L	0.21	0.01	0.68	0.89	0.31	1.36	0.05	0.68	0.05	0.08	0.85	0.81	1.88	0.03	0.38	0.09	0.47	0.89	0.54	0.49	1.47	0.73	0.54	0.76	0.22	0.07	1.15
F4_L	0.58	0.63	0.08	1.27	0.90	1.84	0.89	0.65	0.68	0.35	1.22	0.94	0.92	1.25	0.97	0.39	1.02	0.43	0.51	0.06	1.59	0.44	0.77	0.83	1.11	0.66	0.75
C3_L	2.37	1.21	1.06	0.78	0.56	0.60	0.70	0.66	1.25	0.76	2.30	1.62	0.72	1.45	0.68	0.20	0.22	0.76	0.53	1.73	0.61	0.41	0.69	0.95	0.43	0.21	0.31
C4_L	2.08	1.32	1.45	0.82	0.86	0.22	1.61	1.87	0.43	0.38	2.10	1.84	1.45	0.42	1.85	1.13	0.97	0.82	1.15	1.17	0.49	1.34	1.45	1.18	0.40	1.84	0.87
P3_L	2.62	0.31	2.53	0.94	0.73	0.36	0.59	0.20	0.17	0.22	2.97	1.43	1.96	0.61	1.96	0.85	1.81	0.78	0.59	2.50	1.24	0.36	0.41	0.98	0.45	1.60	0.47
P4_L	0.25	0.86	3.02	1.42	0.89	2.34	0.98	2.67	0.21	0.92	0.83	1.00	0.40	1.60	1.10	1.13	2.38	0.99	0.74	1.76	1.89	0.73	2.14	0.61	1.86	1.05	4.93
O1_L	0.10	0.28	3.55	1.20	0.55	0.40	0.46	0.22	1.21	0.54	0.06	0.39	3.00	1.05	1.43	1.11	3.57	1.03	0.98	2.09	1.22	0.25	0.16	0.59	0.61	1.67	0.64
O2_L	0.41	0.11	0.25	0.08	0.39	0.15	0.09	0.21	0.67	0.38	0.77	0.24	0.52	0.89	0.42	0.34	0.19	0.74	0.77	1.26	1.03	0.34	0.20	0.43	0.67	0.68	0.20
F7_L	2.01	0.57	0.60	0.46	1.07	0.59	0.98	0.17	0.39	0.09	1.90	1.34	0.24	0.74	1.59	0.75	0.43	0.33	0.43	1.08	0.63	0.33	0.70	1.24	0.54	1.77	0.15
F8_L	0.44	0.31	0.48	0.30	2.77	1.24	1.74	0.31	1.17	1.09	1.57	0.55	0.95	0.47	0.31	0.26	0.62	0.22	0.52	0.02	0.28	1.15	0.57	1.47	0.59	2.82	0.09
T7_L	0.67	0.92	0.72	0.08	0.55	1.42	0.13	3.90	0.18	0.88	2.34	1.73	0.97	2.16	0.88	2.82	0.22	0.60	0.88	0.51	0.84	0.26	1.43	1.34	0.69	0.40	3.93
T8_L	2.66	2.43	0.82	1.59	0.12	0.49	0.33	0.26	0.34	0.13	0.57	2.50	1.31	0.48	1.25	0.31	2.49	0.33	0.10	0.13	0.71	0.18	0.47	0.10	1.45	0.37	0.26
P7_L	1.24	0.68	2.79	1.20	1.14	0.85	0.14	0.61	0.47	0.85	3.35	3.71	0.75	0.71	1.04	2.11	1.68	0.44	0.11	2.70	1.51	0.26	0.56	1.06	1.52	1.26	1.08
P8_L	0.08	0.31	1.03	0.82	0.78	0.03	0.59	0.37	0.14	0.73	0.28	0.34	0.56	0.56	0.47	0.68	1.31	0.09	0.32	1.61	1.07	0.77	0.33	0.51	0.92	0.82	1.06
Fz_L	0.76	0.36	0.05	0.83	3.48	2.79	0.47	1.15	0.14	0.10	0.88	0.46	1.94	0.33	0.39	0.12	0.77	1.17	0.66	0.47	1.55	1.95	1.43	0.89	0.46	0.72	1.04
Cz_L	2.52	2.26	0.34	2.27	0.52	0.12	0.71	2.11	1.73	1.07	2.25	3.61	1.94	0.98	1.88	0.94	0.74	0.61	1.16	1.03	0.06	0.59	0.63	0.84	0.60	1.69	0.25
Pz_L	1.22	0.27	3.22	1.38	0.36	0.16	0.32	0.20	0.00	0.16	0.99	0.44	2.42	0.92	1.21	0.16	2.99	0.18	0.24	1.23	1.53	0.16	0.23	0.98	0.84	1.05	0.08
FC1_L	0.89	0.44	0.61	0.86	0.92	0.19	0.68	2.89	0.76	1.85	1.03	1.35	2.35	1.16	2.28	0.49	0.26	0.79	1.94	0.72	0.20	0.31	1.40	0.46	0.19	1.67	1.05
FC2_L	1.64	2.12	0.14	1.24	0.88	1.37	1.81	2.16	0.93	1.00	2.57	1.49	2.02	0.49	2.29	2.09	0.85	0.78	0.56	0.22	0.88	0.41	1.46	0.38	0.45	1.44	1.32
CP1_L	3.28	1.75	2.19	0.97	0.16	0.28	0.01	0.46	0.40	0.45	4.29	2.36	1.84	0.96	0.95	0.11	1.50	0.38	0.43	1.70	0.50	0.10	0.22	1.25	0.59	0.81	0.10
CP2_L	4.22	2.39	2.16	1.89	0.37	0.37	0.58	0.99	0.12	0.51	3.08	2.82	2.15	0.76	1.03	0.72	2.19	0.18	0.33	0.70	1.02	0.54	0.74	1.13	0.53	1.04	0.28
FC5_L	0.30	0.03	0.23	0.03	0.87	0.86	1.59	0.77	2.29	0.19	0.57	0.33	1.00	1.51	1.50	0.36	0.15	1.00	1.93	0.57	0.04	0.92	2.09	2.07	1.32	0.90	1.92
FC6_L	0.34	0.48	0.76	0.38	0.29	0.10	1.46	0.58	0.08	0.43	2.61	1.21	0.74	0.88	1.15	0.94	0.51	0.96	0.80	0.60	0.30	1.26	0.72	1.16	1.73	1.21	0.12
CP5_L	2.42	1.65	2.46	0.85	0.78	1.15	0.51	0.76	0.23	0.11	3.82	1.60	0.75	0.75	1.39	0.88	1.37	1.31	0.94	1.52	1.35	1.01	1.33	0.99	0.51	0.73	0.70
CP6_L	0.10	0.64	2.12	1.61	1.30	1.25	0.71	0.69	0.12	1.22	0.20	4.46	0.41	0.57	1.38	0.81	2.38	1.30	0.30	1.33	1.79	1.94	1.39	0.41	0.80	1.58	3.46

Beta band

	Fp1_S	Fp2_S	F3_S	F4_S	C3_S	C4_S	P3_S	P4_S	O1_S	O2_S	F7_S	F8_S	T7_S	T8_S	P7_S	P8_S	Fz_S	Cz_S	Pz_S	FC1_S	FC2_S	CP1_S	CP2_S	FC5_S	FC6_S	CP5_S	CP6_S
Fp1_L	0.13	0.39	1.14	0.43	0.30	0.25	0.29	0.43	0.65	1.01	1.53	1.20	0.37	0.36	1.42	0.90	0.76	0.50	0.30	0.71	0.71	0.28	0.29	0.13	1.35	0.30	0.67
Fp2_L	0.03	0.44	0.30	0.44	0.81	0.87	1.00	2.33	1.24	1.64	0.85	1.15	0.63	0.10	1.17	1.39	0.15	1.91	1.39	1.08	0.52	1.91	1.80	0.11	1.16	0.90	0.60
F3_L	0.44	0.69	7.25	0.38	1.10	0.67	0.33	1.48	1.36	0.94	1.60	0.95	0.13	0.98	1.76	0.65	0.55	0.07	0.53	0.40	0.27	0.10	0.32	0.59	0.66	0.07	1.54
F4_L	1.80	1.17	2.03	0.66	1.58	0.38	2.16	1.00	3.83	1.44	1.89	0.65	0.16	0.40	0.87	2.31	1.50	1.63	3.07	0.74	0.40	1.77	0.94	0.88	0.30	0.94	1.81
C3_L	0.87	0.57	1.29	0.72	1.54	0.38	1.46	0.75	2.12	0.27	0.28	1.52	1.16	0.28	1.75	1.04	1.04	0.54	0.71	0.68	0.25	1.72	0.51	1.60	0.09	2.34	1.78
C4_L	0.99	1.33	3.22	1.39	0.44	0.00	0.71	0.10	2.45	0.43	2.23	0.59	1.16	0.68	0.94	1.08	1.56	0.78	2.90	1.02	0.63	0.30	0.58	3.69	0.01	0.35	0.61
P3_L	0.14	0.78	1.64	1.13	0.37	1.35	0.73	0.94	0.49	0.18	0.12	0.36	0.51	0.19	1.97	1.66	1.24	1.02	0.51	0.39	0.89	1.32	1.40	1.65	0.48	1.31	1.65
P4_L	0.17	0.31	0.65	0.81	0.99	0.68	1.24	0.18	1.55	0.13	0.20	0.88	2.57	0.66	0.58	0.30	0.65	1.43	0.89	0.01	0.56	0.89	0.71	0.32	0.20	0.57	0.59
O1_L	0.08	0.13	0.54	0.25	0.43	2.89	1.06	0.87	2.13	0.79	0.20	0.27	0.13	0.53	3.00	0.93	0.34	0.61	0.70	0.27	1.04	0.74	0.92	0.63	1.01	1.51	1.31
O2_L	0.19	0.06	0.82	1.04	0.30	0.12	1.54	0.07	1.96	0.76	0.78	0.40	2.29	1.00	0.96	0.07	0.59	0.29	1.23	0.03	0.03	0.42	0.20	0.05	0.69	1.03	0.31
F7_L	0.79	0.57	1.32	1.19	0.69	0.06	0.60	2.68	1.88	0.10	0.81	1.22	1.15	0.47	1.86	2.04	0.44	0.23	1.09	0.69	0.23	0.61	0.26	1.25	1.66	0.55	2.18
F8_L	0.30	0.69	1.78	0.44	2.81	1.10	2.35	1.76	2.65	1.04	0.82	0.77	0.99	1.44	0.76	0.71	0.55	4.98	5.82	2.94	1.13	5.49	1.83	1.23	1.18	0.85	0.57
T7_L	0.81	0.55	0.30	0.77	1.87	0.91	2.93	0.74	0.83	1.39	0.98	0.67	0.51	0.68	1.83	0.47	0.37	1.26	3.51	0.81	1.07	2.40	1.48	0.03	3.69	3.34	0.31
T8_L	0.84	0.75	1.25	0.89	0.95	2.72	0.26	0.71	0.09	1.23	0.55	1.82	0.65	1.65	0.33	0.18	0.93	0.84	0.68	0.43	1.31	0.24	0.39	0.92	2.51	0.83	0.54
P7_L	0.31	0.96	1.77	1.75	0.82	1.09	1.14	1.08	0.75	0.17	0.59	0.96	0.80	0.22	2.68	1.51	1.88	1.15	1.16	0.82	0.70	1.53	1.50	0.60	0.55	1.31	1.32
P8_L	0.38	0.22	0.46	0.67	1.46	0.84	1.50	0.25	0.70	0.06	0.03	0.73	0.67	0.73	0.50	0.89	0.59	1.28	0.32	0.25	0.31	2.86	0.77	1.67	0.39	1.35	0.45
Fz_L	0.74	0.59	1.24	0.19	0.41	0.21	1.52	1.86	2.00	1.11	1.25	0.42	0.16	0.31	1.18	1.73	1.02	0.51	1.88	0.44	0.04	1.04	0.73	0.78	0.37	1.12	0.30
Cz_L	0.62	0.41	1.66	1.14	0.75	0.51	1.19	1.09	2.15	0.47	1.29	1.12	0.12	0.64	1.42	0.75	0.29	0.68	1.54	0.56	0.59	0.85	1.34	3.45	0.02	0.74	0.80
Pz_L	0.04	0.24	1.28	0.05	0.36	0.10	0.60	0.60	0.31	0.43	0.25	0.91	0.19	0.18	0.93	0.15	0.12	0.46	0.56	0.04	0.05	0.53	0.51	1.49	0.13	0.55	0.88
FC1_L	0.30	0.43	4.54	0.19	0.99	0.34	2.28	2.90	3.39	1.38	0.85	0.55	0.46	0.13	2.60	0.28	1.44	0.10	2.31	1.34	0.26	0.91	0.40	1.82	0.03	1.12	1.41
FC2_L	2.11	2.02	1.07	1.13	1.58	0.41	3.29	1.06	5.02	0.79	2.25	0.69	0.72	0.29	1.91	3.55	0.84	1.41	3.22	1.10	0.88	1.91	0.82	3.23	0.05	1.90	0.86
CP1_L	0.11	0.23	1.56	0.69	0.22	0.44	0.57	0.84	0.49	0.38	0.06	0.82	0.21	0.17	1.37	1.98	1.25	0.50	1.13	0.26	0.17	1.54	0.83	1.75	0.19	1.09	1.31
CP2_L	0.18	0.03	1.78	0.66	0.63	0.39	0.82	1.02	1.20	0.46	0.15	0.59	0.66	0.71	0.78	0.75	0.06	0.42	2.31	0.15	0.40	0.70	0.40	2.56	0.15	0.31	2.42
FC5_L	0.60	1.88	0.99	0.95	1.51	0.09	1.16	0.68	0.74	0.69	1.42	0.83	0.11	0.33	1.29	0.09	0.38	0.43	0.07	0.56	0.36	0.19	0.14	0.72	1.09	0.94	1.13
FC6_L	1.20	0.75	2.49	0.15	0.38	0.05	0.66	0.25	3.48	1.00	1.25	0.12	1.33	0.88	1.59	1.56	0.66	0.50	1.52	0.23	0.13	0.35	0.08	3.21	0.44	0.06	0.13
CP5_L	0.47	0.72	0.79	1.07	1.21	1.56	0.97	1.26	0.81	0.14	0.02	0.46	0.53	0.02	1.66	2.58	1.90	0.89	0.85	1.37	1.09	1.53	1.40	0.07	0.57	1.72	2.15
CP6_L	0.40	0.41	1.03	0.74	0.39	0.59	0.75	0.33	1.80	0.30	0.15	0.22	0.67	0.48	0.72	0.91	0.26	0.70	1.29	0.11	0.89	0.51	0.74	0.21	0.18	0.77	1.46

Supplementary Figure 3: F statistic associated to the p values obtained from the multiple linear regression modelling analysis using the full model (Figure 3).

Results from the multiple linear regression modelling
Model 1: listener brain-audio entrainment as predictor

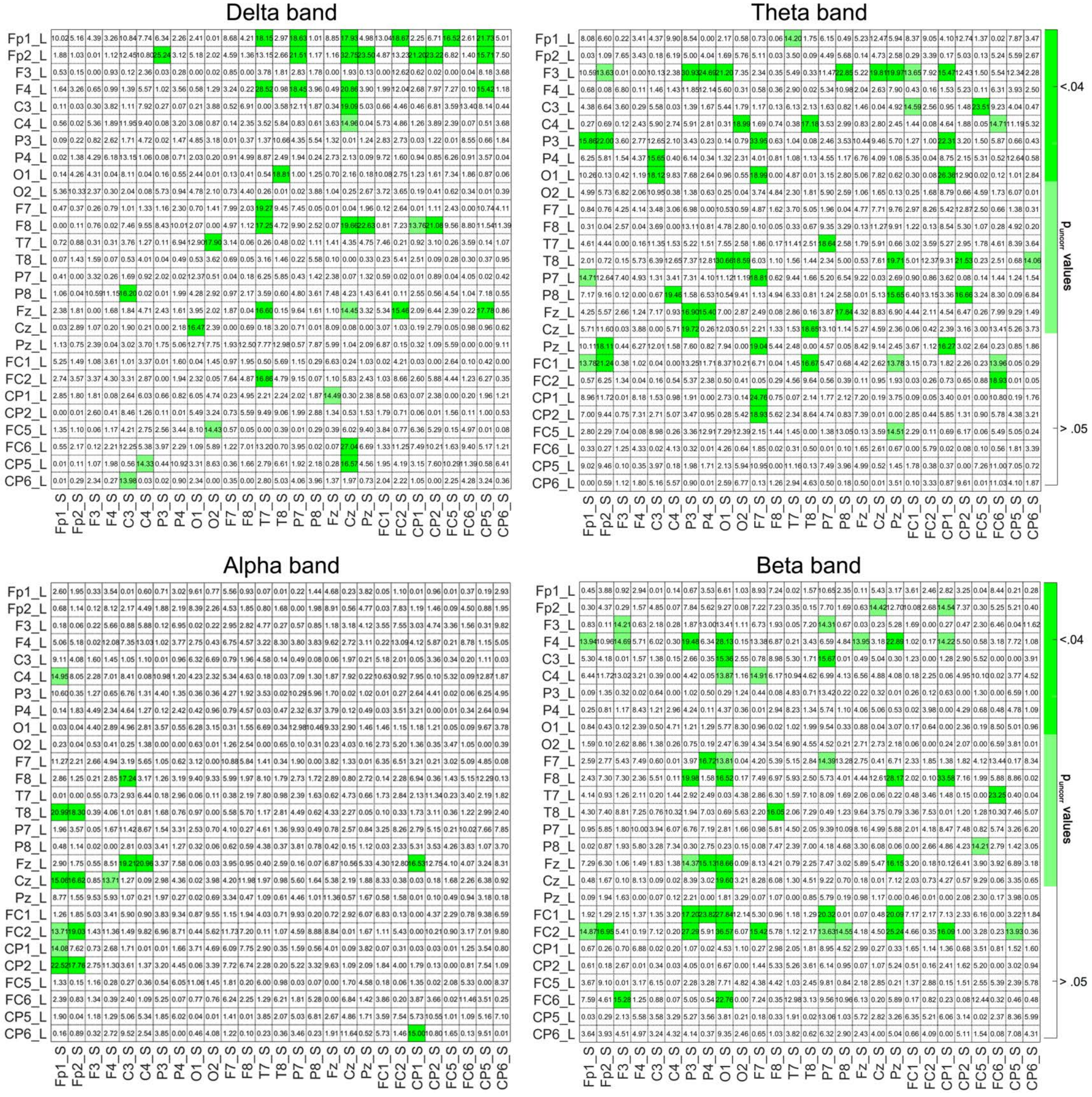

Supplementary Figure 4: Group-level statistical results from the multiple linear regression modelling analysis (Model 1) at the different frequency bands. The matrixes contain the coefficient estimates (R2 values) for a multilinear regression of the brain-to-brain entrainment response using as predictor the brain entrainment to speech of the listeners. Statistically significant percentage of variance explained is indicated by coloring in green the corresponding cells where p<.05. Rows represent the electrodes of the Listener and columns represent the electrodes of the Speaker.

Results from the multiple linear regression modelling
Model 2: speaker brain-audio entrainment as predictor

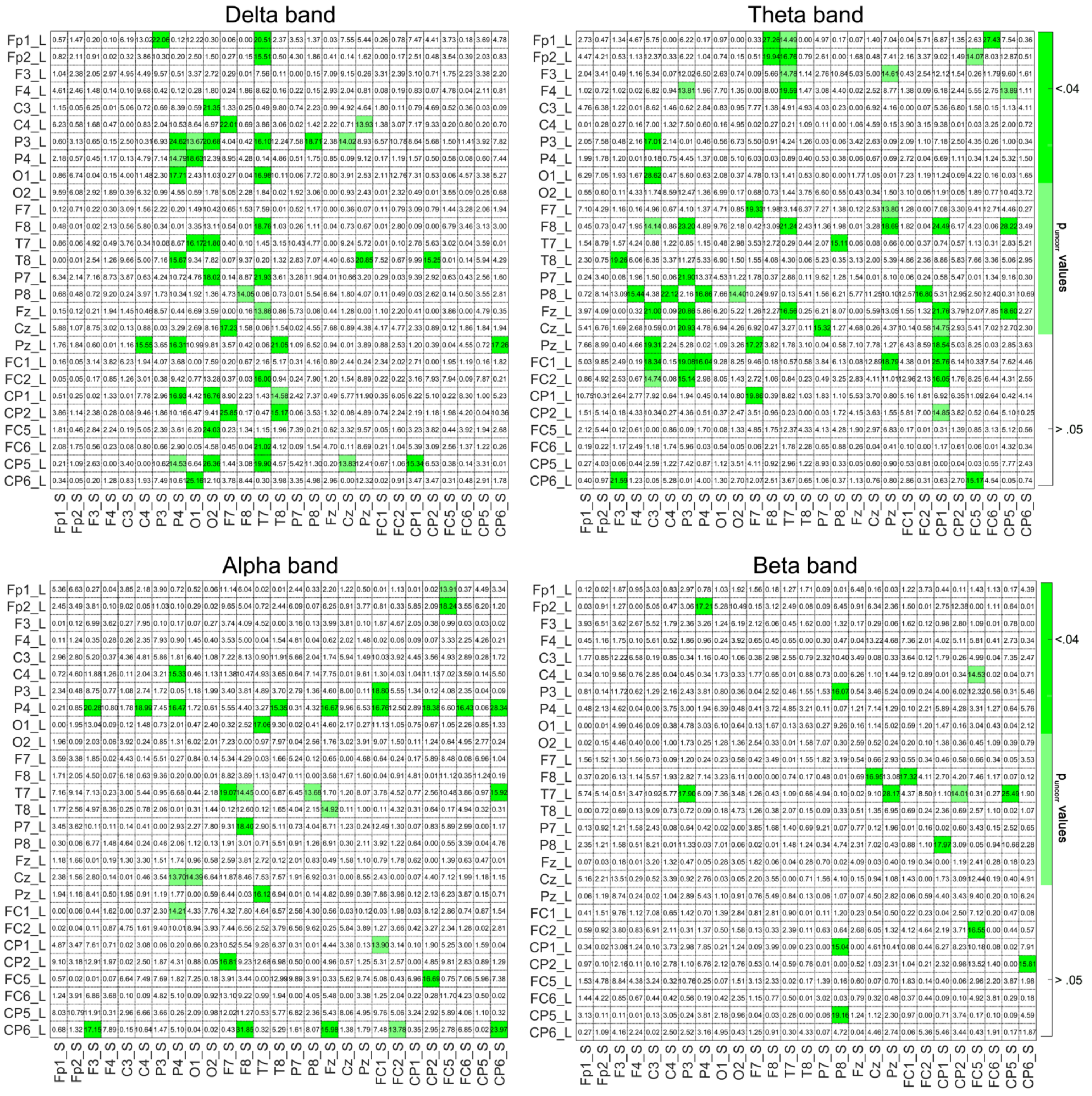

Supplementary Figure 5: Group-level statistical results from the multiple linear regression modelling analysis (Model 2) at the different frequency bands. The matrixes contain the coefficient estimates (R2 values) for a multilinear regression of the brain-to-brain entrainment response using as predictor the brain entrainment to speech of the speakers. Statistically significant percentage of variance explained is indicated by coloring in green the corresponding cells where p<.05. Rows represent the electrodes of the Listener and columns represent the electrodes of the Speaker.

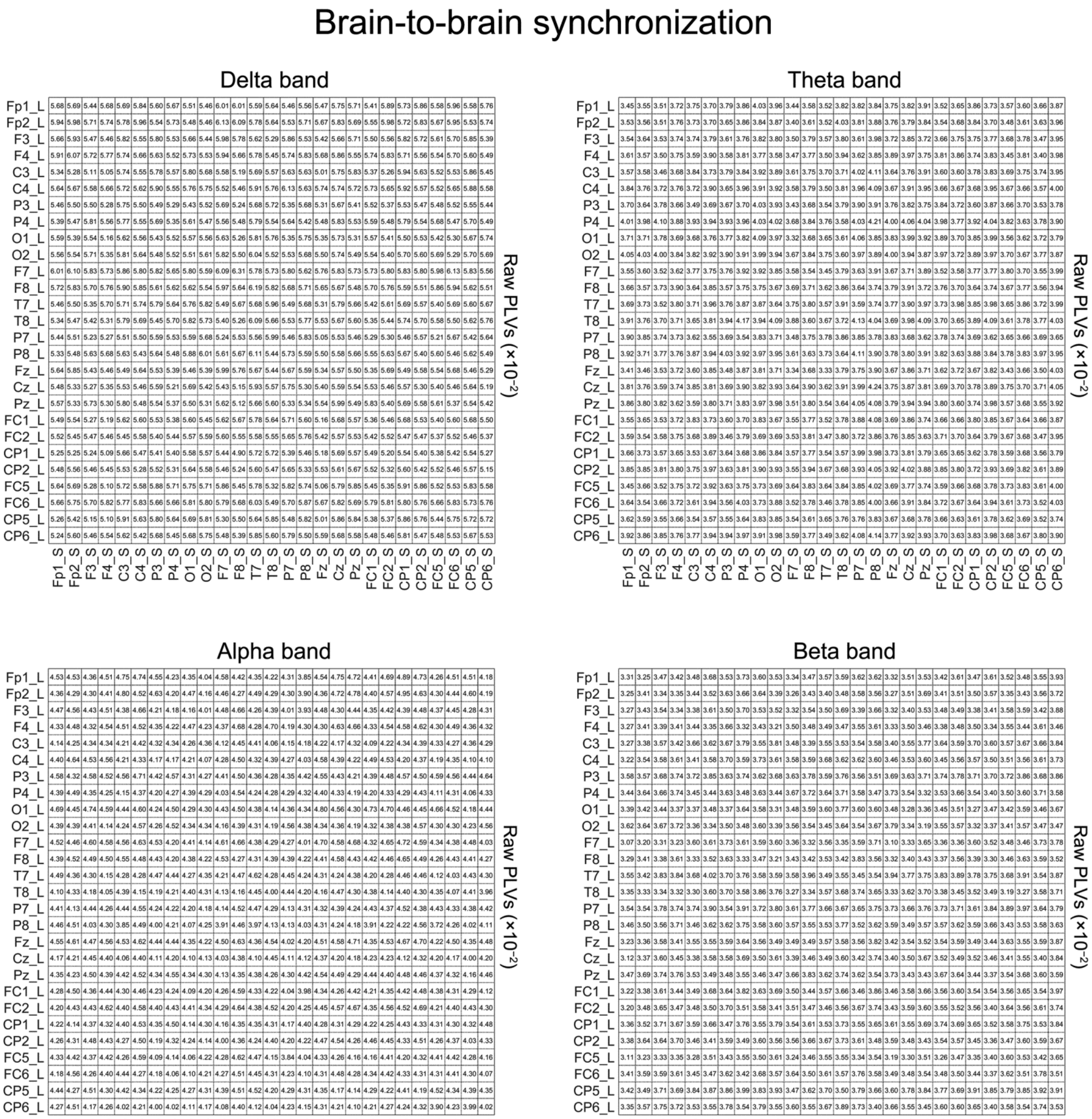

Supplementary Figure 6: Mean raw PLVs (averaged across conditions) obtained from the interbrain synchronization analysis in the delta, theta, alpha and beta band. Rows are the electrodes of the Listener and columns are the electrodes of the Speaker. Each cell corresponds to a listener-speaker's electrode pair. Values are in scientific notation (i.e. PLV $\times 10^{-2}$).

Brain-audio envelope synchronization

Raw PLVs (×10⁻²)

	Listener-Delta	Speaker-Delta	Listener-Theta	Speaker-Theta	Listener-Alpha	Speaker-Alpha	Listener-Beta	Speaker-Beta
Fp1	6.99	8.49	4.20	6.33	4.27	4.48	3.30	3.54
Fp2	7.11	8.26	4.17	6.78	4.27	4.59	3.59	3.45
F3	7.04	8.33	4.17	6.59	4.42	4.55	3.52	3.54
F4	6.97	8.43	4.20	7.09	4.61	4.43	3.47	3.56
C3	6.88	8.29	3.78	6.18	4.40	4.63	3.48	3.60
C4	6.56	8.67	3.99	7.06	4.33	4.82	3.45	3.52
P3	6.35	7.53	3.66	5.61	4.06	4.58	3.57	3.30
P4	6.17	8.02	3.68	5.72	4.31	4.64	3.41	3.47
O1	6.14	8.34	3.71	5.57	4.43	4.67	3.29	3.54
O2	6.09	9.29	3.92	5.33	4.34	4.52	3.57	3.75
F7	6.86	8.81	4.01	6.63	4.25	4.53	3.51	3.61
F8	6.91	8.33	4.13	7.28	4.48	4.78	3.57	3.54
T7	6.74	8.74	3.97	6.36	4.17	4.64	3.68	3.61
T8	7.02	9.37	4.07	7.41	4.29	4.55	3.43	3.38
P7	6.61	8.91	3.71	7.12	4.18	4.77	3.24	3.30
P8	6.07	9.82	3.89	6.36	4.41	4.66	3.52	3.71
Fz	7.00	8.30	4.20	6.79	4.57	4.62	3.43	3.58
Cz	6.79	7.77	4.02	6.68	4.53	4.96	3.44	3.72
Pz	6.14	7.42	3.69	5.53	4.25	4.56	3.43	3.55
FC1	7.03	8.20	4.13	6.55	4.59	4.84	3.43	3.64
FC2	6.85	8.60	4.16	7.14	4.56	4.73	3.63	3.57
CP1	6.35	7.50	3.83	5.96	4.24	4.60	3.39	3.46
CP2	6.21	7.80	3.73	6.09	4.25	4.64	3.34	3.45
FC5	7.45	8.86	4.20	6.78	4.38	4.64	3.63	3.56
FC6	7.03	8.80	4.20	7.48	4.28	4.61	3.45	3.49
CP5	6.46	7.80	3.61	6.38	4.30	4.72	3.47	3.37
CP6	6.21	8.94	3.81	6.45	4.19	4.69	3.14	3.55

Supplementary Figure 7: Mean raw PLVs (averaged across conditions) obtained from the brain-audio envelope synchronization analysis in all the frequency bands. Each cell corresponds to a listener-speaker electrode pair. Rows are the electrodes and columns are the cases: Listener and the Speaker in each frequency band. Values are in scientific notation (i.e. PLV $\times 10^{-2}$).

Supplementary Video 1: Movie showing a 3D topographical representation of statistically significant enhanced alpha band interbrain synchronization patterns between listeners (blue head) and speakers (red head) during oral narratives. Green lines connect those electrode pairs showing an enhanced interbrain synchronization not explained by brain-audio envelope synchronizations.

Supplementary Video 2: Movie showing a 3D topographical representation of statistically significant enhanced beta band interbrain synchronization patterns between listeners (blue head) and speakers (red head) during oral narratives. Green lines connect those electrode pairs showing an enhanced interbrain synchronization not explained by brain-audio envelope synchronizations.
