# Supplementary figures and images for: Brain-to-brain entrainment: EEG interbrain synchronization while speaking and listening
Source: Sci Rep. 2017 Jun 23;7:4190. doi: 10.1038/s41598-017-04464-4 (PMC5482847; doi:10.1038/s41598-017-04464-4)

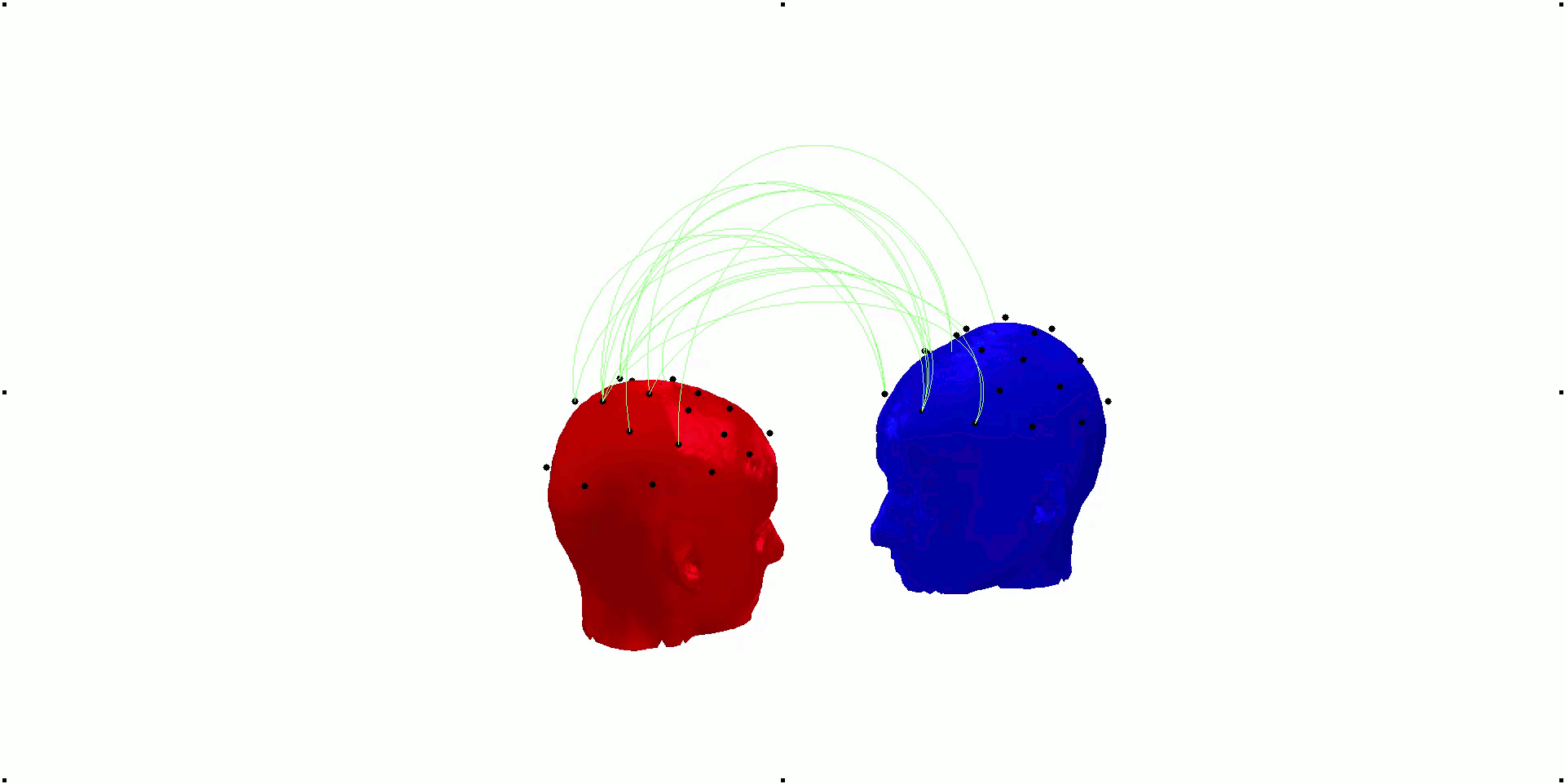

Supplement: Supplementary file 2 — Supplementary Video 1 [file 41598_2017_4464_MOESM2_ESM.gif]

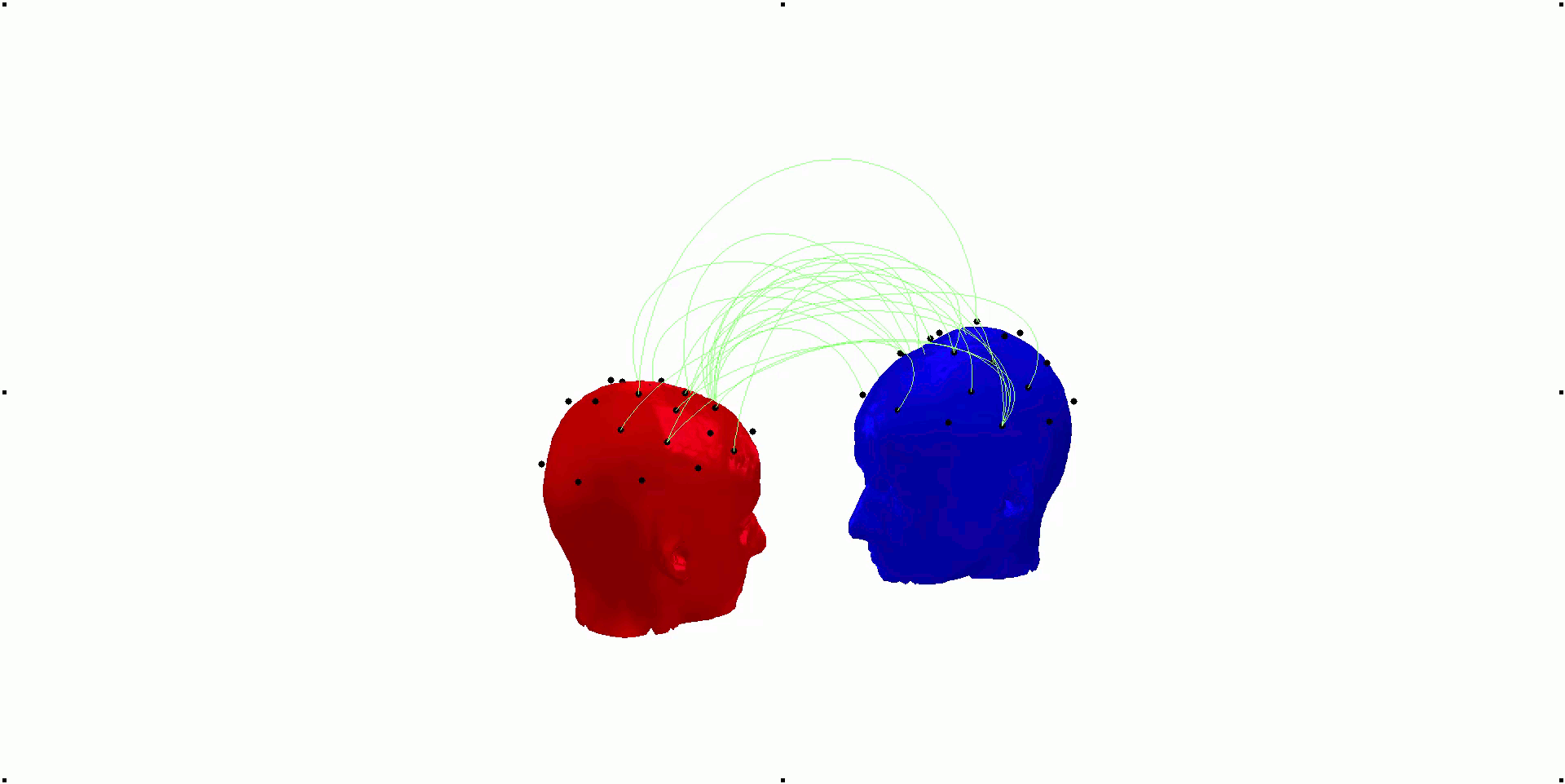

Supplement: Supplementary file 3 — Supplementary Video 2 [file 41598_2017_4464_MOESM3_ESM.gif]
